# Gastric Ulcer Healing Property of *Bryophyllum pinnatum* Leaf Extract in Chronic Model *In Vivo* and Gastroprotective Activity of Its Major Flavonoid

**DOI:** 10.3389/fphar.2021.744192

**Published:** 2021-12-16

**Authors:** Edilane Rodrigues Dantas De Araújo, Gerlane Coelho Bernardo Guerra, Anderson Wilbur Lopes Andrade, Júlia Morais Fernandes, Valéria Costa Da Silva, Emanuella De Aragão Tavares, Aurigena Antunes De Araújo, Raimundo Fernandes de Araújo Júnior, Silvana Maria Zucolotto

**Affiliations:** ^1^ Postgraduate Program in Health Science, Federal University of Rio Grande do Norte (UFRN), Natal, Brazil; ^2^ Department of Biophysics and Pharmacology, Federal University of Rio Grande do Norte, Natal, Brazil; ^3^ Postgraduate Program in Drug Development and Technological Innovation, Federal University of Rio Grande do Norte, Natal, Brazil; ^4^ Postgraduate Program in Pharmaceutical Science, Federal University of Rio Grande do Norte (UFRN), Natal, Brazil; ^5^ Postgraduate Program in Functional and Structural Biology, Department of Morphology, Federal University of Rio Grande do Norte (UFRN), Natal, Brazil; ^6^ Translational Nanobiomaterials and Imaging (TNI) Group, Radiology Department, Leiden University Medical Centrum, Leiden, Netherlands; ^7^ Percuros B.V, Leiden, Netherlands

**Keywords:** gastric ulcer, ulcer healing, gastroprotection, *Bryophyllum pinnatum*, flavonoids, quercetin

## Abstract

Gastric ulcer is a common disease that develops complications such as hemorrhages and perforations when not properly treated. Extended use of drugs in the treatment of this pathology can provoke many adverse effects. Therefore, finding medicinal plants with gastroprotective and mucosal healing properties has gained increasing interest. *Bryophyllum pinnatum* (Crassulaceae), popularly known in Brazil as “*sai*ã*o*” or “*coirama*,” has been used to treat inflammatory disorders. It is rich in flavonoids, and quercetin 3-*O*-α-L-arabinopyranosyl-(1→2)-*O*-α-L-rhamnopyranoside-Bp1 is its major compound. In this study, we aimed to investigate ulcer healing properties of *B. pinnatum* against an acetic acid–induced chronic ulcer model and the gastroprotective activity of Bp1 against gastric lesions induced by ethanol and indomethacin. Ultrafast liquid chromatography was used to quantify the main compounds (mg/g of the extract)—quercetin 3-*O*-α-L-arabinopyranosyl-(1→2)-*O*-α-L-rhamnopyranoside (33.12 ± 0.056), kaempferol 3-*O*-α-L-arabinopyranosyl-(1→2)-*O*-α-L-rhamnopyranoside (3.98 ± 0.049), and quercetin 3-*O*-α-L-rhamnopyranoside (4.26 ± 0.022) and showed good linearity, specificity, selectivity, precision, robustness, and accuracy. *In vivo* studies showed that treatment with the extract at 250 and 500 mg/kg stimulated the healing process in the gastric mucosa with significant ulceration index reduction, followed by improvement in the antioxidant defense system [increased glutathione (GSH) levels, decreased superoxide dismutase upregulation, and malondialdehyde (MDA) levels]. Moreover, the extract decreased interleukin-1β and tumor necrosis factor-*a* levels and myeloperoxidase (MPO) activity, increased interleukin 10 levels, showed a cytoprotective effect in histological analyzes and also downregulated the expression of cyclooxygenase-2 and NF-κB (p65). The pretreatment with Bp1 at a dose of 5 mg/kg reduced gastric lesions in the ethanol and indomethacin models, increased GSH, and decreased MDA levels. In addition, the pretreatment decreased MPO activity, interleukin-1β and tumor necrosis factor-α levels, while also showing a cytoprotective effect in histological analyzes. Our study suggests that treatment with *B. pinnatum* extract showed a higher inhibition percentage than pretreatment with the Bp1. This might in turn suggest that Bp1 has gastroprotective activity, but other compounds can act synergistically, potentiating its effect. We conclude that *B. pinnatum* leaf extract could be a new source of raw material rich in phenolic compounds to be applied in food or medicine.

## Introduction

Gastric ulcer is one of the most common gastrointestinal disorders which affects the worldwide population. It is estimated that approximately 10% of the world population develops this disease, which represents a serious health problem with a large impact on the quality of life of millions of individuals (Havens et al., 2018). Gastric ulcer can penetrate the muscular layers of the stomach, developing acute lesions in the gastric mucosa, and it leads to chronic inflammation when not properly treated and could lead to complications such as hemorrhages and perforations (Tarnawski, 2005; Wang et al., 2018).

The development of gastric ulcer results from an imbalance between some offensive and defensive factors in the stomach. The offensive factors include endogenous pathogenic agents and events [hydrochloric acid, pepsin, lipid peroxidation, and production of reactive oxygen species (ROS)] and also exogenous factors [*Helicobacter pylori* infection, stress, smoking, excessive alcohol consumption, and prolonged use of non–steroid anti-inflammatory drugs (NSAIDs)] (Graham, 2014; Asali et al., 2018). The defensive factors include prostaglandins (PGs), mucin secretion, mucus–bicarbonate barrier, nitric oxide (NO), growth factors, mucosal blood flow, cell regeneration, surface phospholipids, and endogenous antioxidants (Asali et al., 2018).

The main therapeutic intervention for the gastric ulcer is the inhibition of aggressive factors combined with the stimulation of increased defensive factors (Wang et al., 2018). The existing therapeutic drugs for the treatment of this pathology are proton pump inhibitors (PPIs—lansoprazole, omeprazole) and H2-receptor antagonists (H2Ras—ranitidine, famotidine), in addition to antibiotics used to eradicate *H. pylori* (Scally et al., 2018).

However, prolonged use of antisecretory drugs can cause many adverse effects (Kuna et al., 2019; Weltermann et al., 2021). For example, PPIs can cause abdominal pain, nausea, headache, diarrhea, osteoporosis, and fractures, in addition to pneumonia, insomnia, and kidney inflammation (Amang et al., 2017), and be associated with an increased gastric cancer risk (Weltermann et al., 2021). The long-term use of H2Ras can lead to the development of galactorrhea in women, gynecomastia in men, and alteration of the bacterial flora of the gastrointestinal tract (Youn et al., 2018).

The use of PPIs and H2Ras can also lead to rapid tolerance during treatment, followed by a rebound effect of gastric hypersecretion after withdrawal of the drug, and ulcers may develop again (Youn et al., 2018). Therefore, it is necessary to investigate new therapeutic alternatives with fewer adverse effects, which stimulate the ulcer healing process and prevent new relapses. In this context, the use of medicinal plants and derived phytochemicals has gained growing interest in the search for new drugs with less side effects (Bungãu and Popa, 2015) and to combat inflammation and oxidative stress in a more natural, drug-free fashion (de Moraes et al., 2020).

*Bryophyllum pinnatum* (Lam) Pers. [synonym *B. calycinum* and *Kalanchoe pinnata* (Lam) Pers.] species, popularly known in Brazil as “*saião*” or “*coirama*,” is native to Madagascar and belongs to the Crassulaceae family. This family has xeromorphic characteristics which enable its species to adapt to the high incidence of sunlight and water scarcity (Herrera, 2008). In view of this, although *B. pinnatum* is native to Madagascar, it has adapted well to Brazil, mainly to the Caatinga biome (Allorge-Boiteau, 1996; Gehrig et al., 2001), being considered naturalized and not endemic from Brazil (Zappi, 2015).

*B. pinnatum* has been used in traditional medicine in treating inflammatory disorders (Amaral et al., 2005), gastritis, and ulcer problems (The Plant List, 2010; Fernandes et al., 2019). Furthermore, in our previous study, it was observed that the leaf extracts of this species showed gastroprotective effects in acute gastric lesions induced by ethanol and indomethacin in rats (Araújo et al., 2018). The literature describes the presence of phenolic compounds in the leaves of this species, highlighting the presence of flavonoids. Glycosides derived from quercetin, patuletin, eupafolin, and kaempferol have been identified in *B. pinnatum* leaf extract (Muzitano et al., 2006; Afzal et al., 2012; Fernandes et al., 2016; Araújo et al., 2018).

Quercetin3-*O*-α-L-arabinopyranosyl-(1→2)-*O*-α-L-rhamnopyranoside is a flavonoid isolated from *B. pinnatum* leaves, being considered the major compound and marker for this species (Nascimento et al., 2018; Fernandes et al., 2021). This compound contains a rare sugar moiety and has shown anti-inflammatory and leishmanicidal activities (Muzitano et al., 2006; Ferreira et al., 2014), therefore is a possible marker for the species.

Quercetin, one such member of the flavonoid family, is one of the most prominent dietary antioxidants, more active than the well-known vitamins E and C antioxidants (Russo et al., 2012; Farzaei et al., 2015). Quercetin has been reported to have protective effects against various diseases such as osteoporosis, certain cancers, pulmonary and cardiovascular diseases, and aging and has been reported to prevent gut microbiota dysbiosis, in addition to presenting antioxidant and anti-inflammatory activities (Bhutada et al., 2010; Kumari et al., 2010; Russo et al., 2012; Wu et al., 2012; Cardona et al., 2013; Etxeberria et al., 2015; Farzaei et al., 2015).

Considering the promising preliminary results with *B. pinnatum* leaf extract in acute gastric lesions induced by ethanol and indomethacin and the relevant therapeutic potential of this species, this work has the objective to evaluate ulcer healing properties of *B. pinnatum* leaf extract against an acetic acid–induced chronic ulcer model in rats and the gastroprotective activity of quercetin 3-*O*-α-L-arabinopyranosyl-(1→2)-*O*-α-L-rhamnopyranoside against gastric lesions induced by ethanol and indomethacin in mice, and to quantify the flavonoid content by ultrafast liquid chromatography analysis.

## Materials and Methods

### Plant Material and Reagents

*B. pinnatum* leaves were collected from the “Escola Agrícola de Jundiaí” in Macaíba city, Rio Grande do Norte state, Brazil, in the February of 2018. The botanical identification voucher specimen (No. 57335) was deposited at the herbarium of the Bioscience Center of the Federal University of Rio Grande do Norte. The collection was authorized by the Brazilian Authorization and Biodiversity Information System (SISBIO process No. 35017), and the research was authorized by the National System for the Management of Genetic Heritage and Associated Traditional Knowledge (SISGEN process no. A7EA798).

Most of the chemicals used were purchased from Sigma-Aldrich (São Paulo, Brazil, Madrid, Spain), unless otherwise stated. Quercetin 3-*O*-α-L-arabinopyranosyl-(1→2)-*O*-α-L-rhamnopyranoside (Bp1) (99.3% purity), kaempferol 3-*O*-α-L-arabinopyranosyl-(1→2)-*O*-α-L-rhamnopyranoside (Bp2) (98.9% purity), and quercetin 3-*O*-α-L-rhamnopyranoside (Bp3) (99.6% purity) were previously isolated from *B. pinnatum* and identified by nuclear magnetic resonance (NMR) and mass spectrometry (MS). The purity of the isolated compounds was determined by software of ultrafast liquid chromatography (UFLC) coupled with a diode-array detector (DAD) (Shimadzu Model LC-20AD, with DAD detector model SPD-M20A). NMR analysis of each sample also showed integration signals related to one compound (results not shown).

### Preparation of the *Bryophyllum pinnatum* Leaf Extract

Fresh *B. pinnatum* leaves (4.3 kg) were processed by turbo extraction with water in a proportion of 1:1 (w/v) in an industrial blender for 5 min to obtain the *B. pinnatum* leaf extract. The extract was sieved and concentrated on a rotoevaporator (model V-700, Buchi, Flawil, Switzerland) and freeze-dried (extraction yield of 4.40%). For *in vivo* assays, the extract was solubilized in distilled water.

### Isolation and Identification of Main Flavonoids From *B. pinnatum*

The main flavonoids from *B. pinnatum* were previously isolated and identified by Fernandes et al. (2021). Three compounds [quercetin 3-*O*-α-L-arabinopyranosyl-(1→2)-*O*-α-L-rhamnopyranoside (Bp1), kaempferol 3-*O*-α-L-arabinopyranosyl-(1→2)-*O*-α-L-rhamnopyranoside (Bp2), and quercetin 3-*O*-α-L-rhamnopyranoside (Bp3)] were efficiently purified; all of them as amorphous yellow powder. The compounds yield provided 149.4 mg of Bp1, 14.1 mg of Bp2, and 6.7 mg of Bp3. Details about the isolation and structural elucidation can be obtained in Fernandes et al. (2021).

### Quantitative Analyses of Flavonoids Bp1, Bp2, and Bp3 by UFLC-DAD

*B. pinnatum* extract was analyzed by UFLC-DAD (Shimadzu Model LC-20AD, Kyoto, Japan, with DAD detector model SPD-M20A and LabSolutions software, Shimadzu, Kyoto, Japan). The method used was validated according to Brazilian legislation No. 166 from July 24, 2017 (Brazil, 2017), which is aligned with the Guideline Q2 (R1) of the International Conference on Harmonisation of Technical Requirements for Registration of Pharmaceuticals for Human Use (ICH, 2005). The following parameters were analyzed: selectivity, linearity, limits of detection (LOD) and limits of quantification (LOQ), precision, accuracy, and robustness. Prior to starting the validation, the system suitability of the developed method was verified according to the parameters established in the United States Pharmacopeia to guarantee resolution (R > 1.5), column efficiency (*p* > 2,000), tailing factor (T ≤ 2.0), and capacity factor (k′ ≥ 2.0) for each peak to be subsequently quantified (USP, 2003). All analyses were performed in triplicate, and the relative standard deviation was calculated. The samples (*B. pinnatum* extract and Bp1) were resuspended in 1:1 methanol:water (v/v), and the extract was analyzed at the concentration of 2 mg/ml and Bp1, Bp2, and Bp3 at the concentration of 200 μg/ml. A Phenomenex Kinetex Core-Shell RP-18 column (150 mm × 4.6 mm, 2.6 mm particle size) equipped with a Phenomenex security guard column (4.0 mm × 2.0 mm ID) was used. The eluents were 1) trifluoroacetic acid 0.3% and 2) acetonitrile. The following gradient (v/v) was applied: 7–15% B, 0–3 min; 15–20% B, 3–12 min; 20–22% B, 12–30 min; with 30 min of analysis time. Flow elution was 0.7 ml/min, and 12 ml of each sample was injected. The UV-DAD detector was programmed for wavelength 200–500 nm, and the chromatogram was plotted at 254 and 340 nm. *B. pinnatum* is a plant rich in flavonoids, therefore the chromatogram was analyzed at UV 340 nm and also recorded at UV 254 nm to verify the presence or absence of other possible secondary metabolites present in the extract (Aparicio and Harwood, 2013). UFLC-grade acetonitrile and trifluoracetic acid were purchased from J. T. Baker (Brazil). Water was purified with a MilliQ system (Millipore, Burlington, Massachusetts, United States). The samples and solvents were filtrated through a membrane (pore size of 0.45 m) and degassed. Each sample was analyzed in triplicate.

### *In Vivo* Studies

Female Wistar rats (180 ± 20 g, 6–8 weeks old) were used for the evaluation of ulcer healing in the acetic acid–induced chronic ulcer, and female Swiss albino mice (25 ± 10 g, 6–8 weeks old) were used for the gastroprotective activity in the models of induction of gastric lesions by ethanol and indomethacin. All animals were raised in accordance with the National Institute of Health Guide for Laboratory Animals. Rodents were acclimated for 7 days prior to experimentation, were housed under standard environment conditions at 20–25°C and 12-h dark/light cycle, and had free access to potable water (*ad libitum*) and standard food. These procedures were approved by the Ethics Committee of Laboratory Animals of the Federal University of Rio Grande do Norte (CEUA No. 029.047/2017).

### Evaluation of Ulcer Healing Property of *B. pinnatum* Leaf Extract

#### Acetic Acid–Induced Chronic Gastric Ulcer

Chronic gastric ulcers were induced according to the method described by Okabe et al. (2005). Female Wistar rats were divided randomly into six experimental groups with six animals in each group (*n* = 6) and were fasted 18 h before the surgical procedure for induction of chronic gastric ulcer. Under anesthesia with ketamine (50 mg/kg) and xylazine (5 mg/kg) by intraperitoneal (IP) injection, laparotomy was performed through a midline epigastric incision; the stomach was exposed and 80% acetic acid (0.03 ml) was instilled for 1 min into the subserosal layer of the glandular portion, using a microsyringe (0.05 ml) and a cannula (6 mm of diameter). Afterward, the fluid was aspirated off carefully and the area that remained in contact with the acid was gently rinsed with sterile saline solution. Then, the stomach was located to the anatomical region, and the anterior wall of the abdomen closed by continuous 2.0 silk suture. Forty-eight hours after ulcer induction, the rats were orally treated with vehicle (distilled water, 10 ml/kg), lansoprazole (30 mg/kg), and *B. pinnatum* leaf extract in the doses of 125, 250, and 500 mg/kg, respectively, twice a day for 7 days. On the following day, the animals were euthanatized by cervical dislocation under anesthesia [ketamine (80 mg/kg) and xylazine (10 mg/kg, IP)], and the stomachs were removed and opened along the greatest curvature, washed with sterile saline solution, and then macroscopically evaluated for measuring the injured areas. The stomach samples were stored at −80°C for analyses.

### Gastroprotective Activity of Quercetin 3-*O*-α-L-Arabinopyranosyl-(1→2)-*O*-α-L-Rhamnopyranoside (Bp1)

#### Ethanol-Induced Gastric Lesions

The induction of gastric lesion by ethanol was adapted from the method of Hollander and Tarnawski (1986). After 24 h of fasting, the mice (*n* = 7/group) were orally pretreated at 24 and 1 h prior to induction. The Healthy Group and the Gastric Lesion Group received the vehicle (distilled water, 10 ml/kg) orally. The group pretreated with the standard drug received an oral dose of the 50 mg/kg ranitidine, and the groups pretreated with Bp1 received an oral dose of 2.5, 5, and 10 mg/kg. After 1 h of the pretreatment, all animals except those in the Healthy Group received an oral dose of the 0.5 ml/100 g absolute ethanol PA. The mice were euthanized 1 h later by cervical dislocation under anesthesia [ketamine (80 mg/kg) and xylazine (10 mg/kg, IP)], the stomachs were removed and opened along the greatest curvature, washed with sterile saline solution, and then macroscopically evaluated for measuring the injured areas. The stomach samples were stored at −80°C for analyses.

#### Indomethacin-Induced Gastric Lesions

The induction of gastric lesion by indomethacin was adapted from the method of Kakub and Gulfraz (2007). After 24 h of fasting, the mice (*n* = 7/group) were orally pretreated at 24 and 1 h prior to induction. The Healthy Group and the Gastric Lesion Group received the vehicle (distilled water, 10 ml/kg) orally. The group pretreated with the standard drug received an oral dose at 50 mg/kg ranitidine, and the groups pretreated with Bp1 received an oral dose at 2.5, 5, and 10 mg/kg. After 1 h of the pretreatment, all animals except those in the Healthy Group received an oral dose of the 40 mg/kg indomethacin. The mice were euthanized 6 h later by cervical dislocation under anesthesia with ketamine (80 mg/kg) and xylazine (10 mg/kg, IP), the stomachs were removed and opened along the greatest curvature, washed with sterile saline solution, and then macroscopically evaluated for measuring the injured areas. The stomach samples were stored at −80°C for analyses.

### Macroscopic Stomach Lesion Assessment

In order to determine the Gastric Lesion Index (GLI), the scores were attributed as described by Magistretti et al. (1988) with adaptations. The extent of gastric damage was quantified by measuring the area of gastric lesions. The stomach was thoroughly rinsed with sterile saline solution to remove any contents. Then, the stomach samples were photographed. The digital photos were used for the determination of the total stomach area (mm^2^) and area of the gastric lesions (mm^2^) using ImageJ 1.48d software (National Institute of Health, Bethesda, MD, United States). For each stomach, the sum of the areas of all forms of gastric lesions was recorded. The GLI and Percentage Inhibition (I%) were calculated according to the following equations:

$$\mathrm{GLI=}\left[\mathrm{lesion}\;\mathrm{area}\;\left(mm^{2}\right)\mathrm{/total}\;\mathrm{stomach}\;\mathrm{area}\left(mm^{2}\right)\right]\mathrm{\times 100},$$

I%=[(GLI gastric lesiongroup- GLI pre-treated group)/GLI gastric lesion contro]×100.

### Nonprotein Sulfhydryls Assay

Glutathione (GSH) levels in gastric tissue were measured as a marker of antioxidant activity. The stomach samples were harvested and stored at −80°C until required for the assay. The gastric tissue homogenate (0.25 ml 5% tissue solution prepared in 0.02 M ethylenediaminetetraacetic acid) was added to 320 µl distilled water and 80 µl 50% trichloroacetic acid. The samples were centrifuged at 3,000 rpm and 4°C for 15 min. Then, 400 µl supernatant was added to 800 µl 0.4 M Tris buffer (pH 8.9) and 20 µl 0.01 M 5,5′-dithio-bis-[2-nitrobenzoic acid]. Sample absorbance was measured at 420 nm. The results were reported as units of GSH per milligram of tissue.

### Malondialdehyde Assay

Malondialdehyde (MDA) content was measured by the assay described by Esterbauer and Cheeseman (1990). The stomach samples were suspended in buffer Tris hydrochloric acid (HCl) 1:5 (w/v), pH = 7.4, and minced with scissors for 15 s on an ice-cold plate. The resulting suspension was homogenized for 2 min with an automatic Potter homogenizer and centrifuged at 2,500 ×*g* at 4°C for 10 min. The supernatants were assayed to determine the MDA content. Chromogen reagent 1-methyl-2-phenylindole was used in the reaction. The absorbance was measured at 586 nm and was calculated by interpolation in standard curve with 1,1,3,3 tetraethoxypropane (10 mM), hydrolyzed during incubation with HCl at 45°C for 40 min. The results are expressed as nanomoles of MDA per gram of tissue.

### Myeloperoxidase Activity

The stomach samples were harvested as described above and stored at −80°C until required for the assay. After homogenization in hexadecyltrimethylammonium bromide 0.5% (pH = 6.0), and centrifugation (2000 ×*g* for 20 min at 4°C), myeloperoxidase (MPO) activity was determined by a previously described colorimetric method (Krawisz et al., 1984). *o*-Dianisidine dihydrochloride staining reagent, potassium phosphate buffer (PB), and 0.05% hydrogen peroxide at 1% were used in this reaction. The absorbance of the samples was determined at 450 nm in a 96-well microplates reader. The results were expressed as the amount of enzyme that degrades 1 μmol ml^−1^ of peroxide at 25°C and reported as units of MPO per gram of tissue.

### Interleukin-1β, Tumor Necrosis Factor-α, and Interleukin-10 Assays

The tissue was homogenized and processed as described by Safieh-Garabedian et al. (1995). The levels of interleukin-1β (IL-1β) [detection range: 62.5–4,000 pg/ml; sensitivity or lower limit of detection (LLD): 12.5 ng/ml of recombinant mouse IL-1β], tumor necrosis factor-α (TNF-α) (detection range: 62.5–4,000 pg/ml; sensitivity or LLD: 50 ng/ml of recombinant mouse TNF-α), and interleukin-10 (IL-10) (detection range: 62.5–4,000 pg/ml; sensitivity or LLD: 50 ng/ml of recombinant mouse IL-10) in the stomach samples were determined with a commercial ELISA kit (R&D Systems, Minneapolis, MN, United States), as previously described. All samples were within the wavelength used in UV-VIS spectrophotometry (absorbance measured at 490 nm).

### Histopathology Analysis

The specimens of the gastric walls for all the animal groups were fixed in 10% buffered formalin solution and processed by light microscopy using the paraffin slice technique. The sections with 4 µm thickness were stained with hematoxylin and eosin (H&E) stain for histological evaluation. The criteria for evaluating gastric lesions and leukocyte infiltration and distribution were carried out according to the parameters described by Dokmeci et al. (2005). Reported histopathological analyses were independently performed by two pathologists, blinded to the group identity.

### Immunohistochemical Staining of Superoxide Dismutase, Cyclooxygenase-2, and Nuclear Factor-Kappa B-p65

Thin stomach sections (3 µm) were obtained from each group of the chronic ulcer experiment (Healthy Group, Gastric Lesion Group, lansoprazole, and dose of 250 mg/kg and 500 mg/kg of *B. pinnatum* leaf extract) with a microtome and transferred to gelatine-coated slides. Each tissue section was then deparaffinized and rehydrated. The stomach tissue slices were washed with 0.3% Triton X-100 in PB and quenched with endogenous peroxidase (3% hydrogen peroxide). The tissue sections were incubated overnight at 4°C with primary antibodies (Santa Cruz Biotechnology, Santa Cruz, CA, United States) against nuclear factor-kappa B-p65, cyclooxygenase-2 (COX-2), and superoxide dismutase (SOD) primary antibodies (Spring-Abcam, MA, United States). Dilution tests (three dilutions) were performed with all antibodies to identify the 1:100, 1:500, and 1:100 dilutions as appropriate, respectively. The slices were washed with PB and incubated with a streptavidin/HRP-conjugated secondary antibody (Biocare Medical, Concord, CA, United States) for 30 min. Immunoreactivity to the various proteins was visualized with a colorimetric-based detection kit following the protocol provided by the manufacturer (TrekAvidin-HRP Label + Kit from Biocare Medical, Dako, CA, United States). The sections were counterstained with hematoxylin. Known positive controls and negative controls were included in each sample set. Planimetry microscopy (Nikon E200 LED, Morphology Department/UFRN) with a high-power objective (40×) was utilized to score the intensity of cell immunostaining, according to the methodology used by Araújo Júnior et al. (2014).

### Statistical Analysis

All values are reported as the mean ± standard mean error or as mean ± standard deviation and were analyzed by one-way analysis of variance (ANOVA) followed by Tukey or Dunnett post-hoc test for multiple comparisons. Nonparametric data (score) are expressed as the median (range) and were analyzed using the Mann–Whitney test. All statistical analyzes were performed using GraphPad 7.0 software (Graph-Pad Software Inc., La Jolla, CA, United States), and the statistical significance was set at *p* < 0.05.

## Results

### Quantitative Analyses of Flavonoids Bp1, Bp2, and Bp3 by UFLC-DAD

**FIGURE 1:**
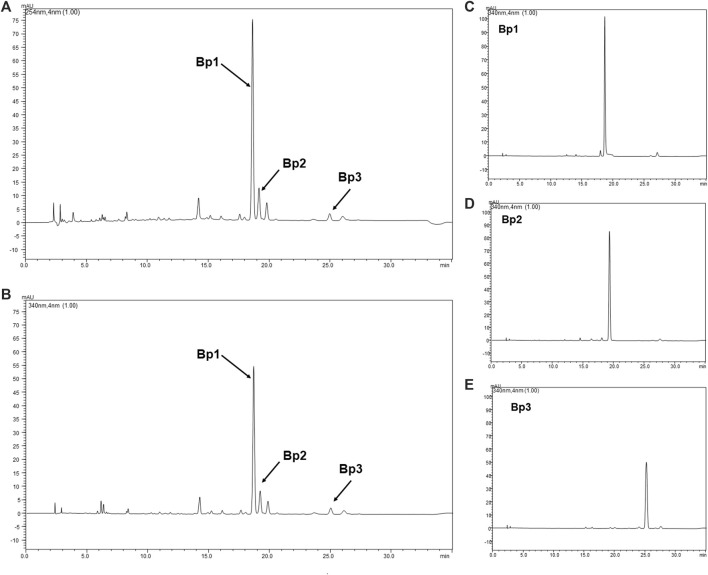
Chromatogram of *B. pinnatum* extract at UV 254 nm **(A)** and 340 nm **(B)** and chromatogram at UV 340 nm of Bp1 **(C)**, Bp2 **(D)**, and Bp3 **(E)**. Stationary phase: C18 Phenomenex (150 mm × 4.6 mm, 2.6 mm) equipped with a Phenomenex security guard column (4.0 mm × 2.0 mm ID). Mobile phase: trifluoroacetic acid 0.3% and acetonitrile; gradient: 7–15% B, 0–3 min; 15–20% B, 3–12 min; 20–22% B, 12–30 min; flow elution 0.7 ml/min; detection 254 and 340 nm.

**FIGURE 2:**
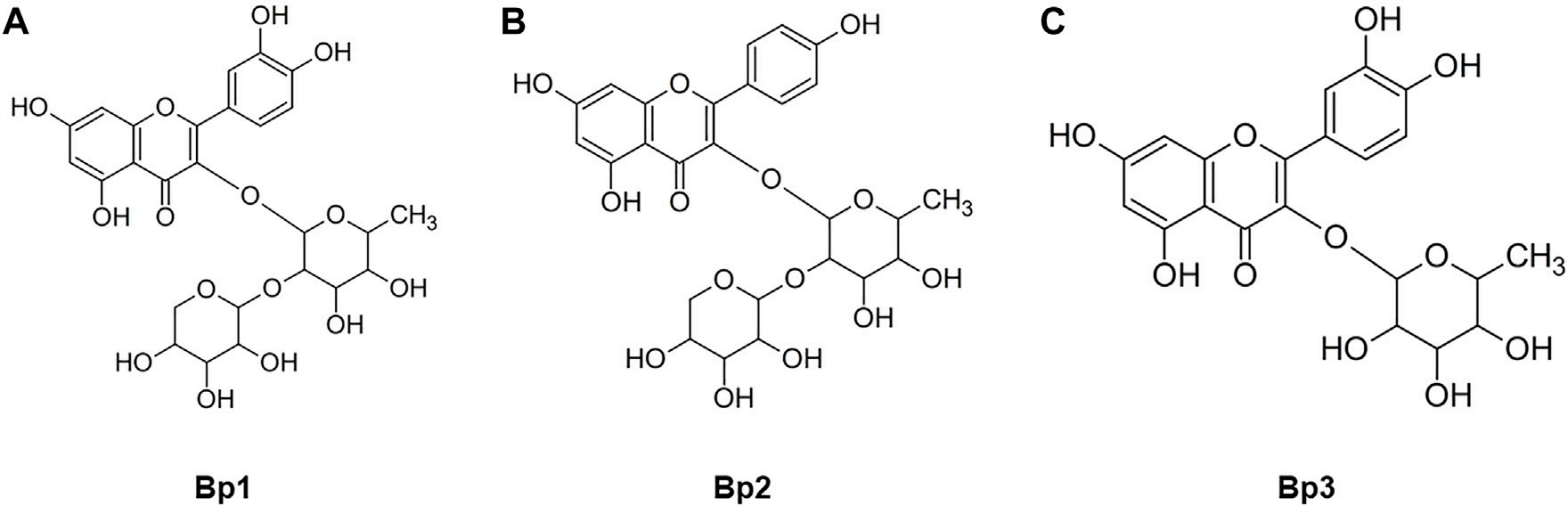
Structures of the flavonoids **(A)** quercetin 3-*O*-α-L-arabinopyranosyl-(1→2)-*O*-α-L-rhamnopyranoside (Bp1), **(B)** kaempferol 3-*O*-α-L-arabinopyranosyl-(1→2)-*O*-α-L-rhamnopyranoside (Bp2), and **(C)** quercetin 3-*O*-α-rhamnopyranoside (Bp3) isolated from *B. pinnatum* leaves.

A *B. pinnatum* leaf extract chromatogram is shown in Figure 1 with a major peak and other peaks at UV 254 (Figure 1A) and 340 nm (Figure 1B), corresponding to the flavonoids: Bp1 (*Rt* 19.20 ± 0.26) (Figure 1C), Bp2 (*Rt* 19.87 ± 0.28) (Figure 1D), and Bp3 (*Rt* 25.58 ± 0.20) (Figure 1E). Their UV spectra are similar to those of quercetin (256 and 265 nm—II band, and 355 nm—I band). The structures of the Bp1, Bp2, and Bp3 flavonoids are shown in Figures 2A–C, respectively. Extract peaks were identified by comparing retention times, UV spectra data, and increasing peak areas by co-injection (extract + standard solutions, 1:1, v/v). The peaks were found to have already been identified and previously described by our research group (Fernandes et al., 2016; Andrade et al., 2020; Fernandes et al., 2021), but this is the first time that the major flavonoids have been quantified and correlated with the pharmacological activity. In a previous study, the major flavonoids identified in *B. pinnatum* by high-performance liquid chromatography coupled with DAD-MS/MS were flavonoids-*O*-glycosides derived from aglicones eupafolin, quercetin, and kaempferol. In this work, the major flavonoid (Bp1) and two other compounds (Bp2 and Bp3) were quantified as possible analytical markers of this species. The UFLC-DAD method for quantifying flavonoids indicated good linearity (*r* > 0.9997—Bp1; *r* > 0.9996—Bp2; *r* > 0.9992—Bp3), specificity, selectivity, precision, robustness, and accuracy (Table 1). The Bp1, Bp2, and Bp3 contents were 33.12 ± 0.056, 3.98 ± 0.049, and 4.26 ± 0.022 mg, respectively, per gram of extract (Table 1).

**TABLE 1:** Validation parameters of the analytical method to quantify the content of Bp1, Bp2, and Bp3 in *B. pinnatum* extract at UV 340 nm.

Compounds	System suitability parameters^a^			Selectivity parameters				Linearity	LOD^c^ (μg/ml)	LOQ^c^ (μg/ml	Content (mg/g)
	R	T	P	k′	Peak purity	*Rt* (min.)	r	Calibration curve^b^			
Bp1	1.68 ± 0.023	1.33 ± 0.201	51413,23 ± 435.631	7.77 ± 0.176	0.99 ± 0.01	19.20 ± 0.26	0.9997	y = 457720 × —11163	0.0001713	0.0005191	33.12 ± 0.056
Bp2	2.05 ± 0.019	1.19 ± 0.027	41901,22 ± 298.611	7.83 ± 0.366	0.98 ± 0.005	19.87 ± 0.28	0.9996	y = 92220 × —698.53	0.0007442	0.0022553	3.98 ± 0.049
Bp3	2.61 ± 0.038	1.03 ± 0.004	49,248 ± 343.211	10.49 ± 0.328	0.99 ± 0.006	25.58 ± 0.20	0.9992	y = 194,10 × + 198.17	0.0007428	0.0006598	4.26 ± 0.022
**Compounds**		**Intermediate precision** ^**d**^			**Repeatability** ^**e**^				**Accuracy** ^**f**^		
		**50%**	**100%**	**150%**	**50%**	**100%**	**150%**		**50%**	**100%**	**150%**
Bp1	RSD (%)	3.49	3.24	0.48	0.97	0.22	0.50	Recovery (%)	111.92	98.85	90.99
Bp2		1.71	1.11	1.05	1.07	2.19	0.37		101.07	89.70	102.16
Bp3		3.28	3.29	1.47	2.01	2.59	1.68		98.37	91.88	99.03

R, resolution; T, tail factor; P, column efficiency or theoretical plates; k′, capacity factor; Rt, retention time; r, coefficient of correlation; RSD, relative standard deviation.

a Data obtained through the analysis of peak of compounds Bp1, Bp2, and Bp3 from *B. pinnatum* extract.

b y = peak area and x = concentration (mg/ml).

c LOD = 3.3R/S and LOQ = 10R/S: where R and S are the residual standard deviation of the regression line and slope of the calibration curve, respectively.

d RSD of the mean of the samples analyzed on three different days (*n* = 3).

e RSD of the mean of the samples analyzed on the same day (*n* = 3).

f Accuracy determined by the recovery method were added quantities known of reference substance (Bp1, Bp2, and Bp3) to sample (*B. pinnatum* extract at 2 mg/ml).

## *In Vivo* Studies

### Evaluation of Ulcer Healing Property of *Bryophyllum pinnatum* Extract

#### Macroscopic Stomach Lesion Assessment

**FIGURE 3:**
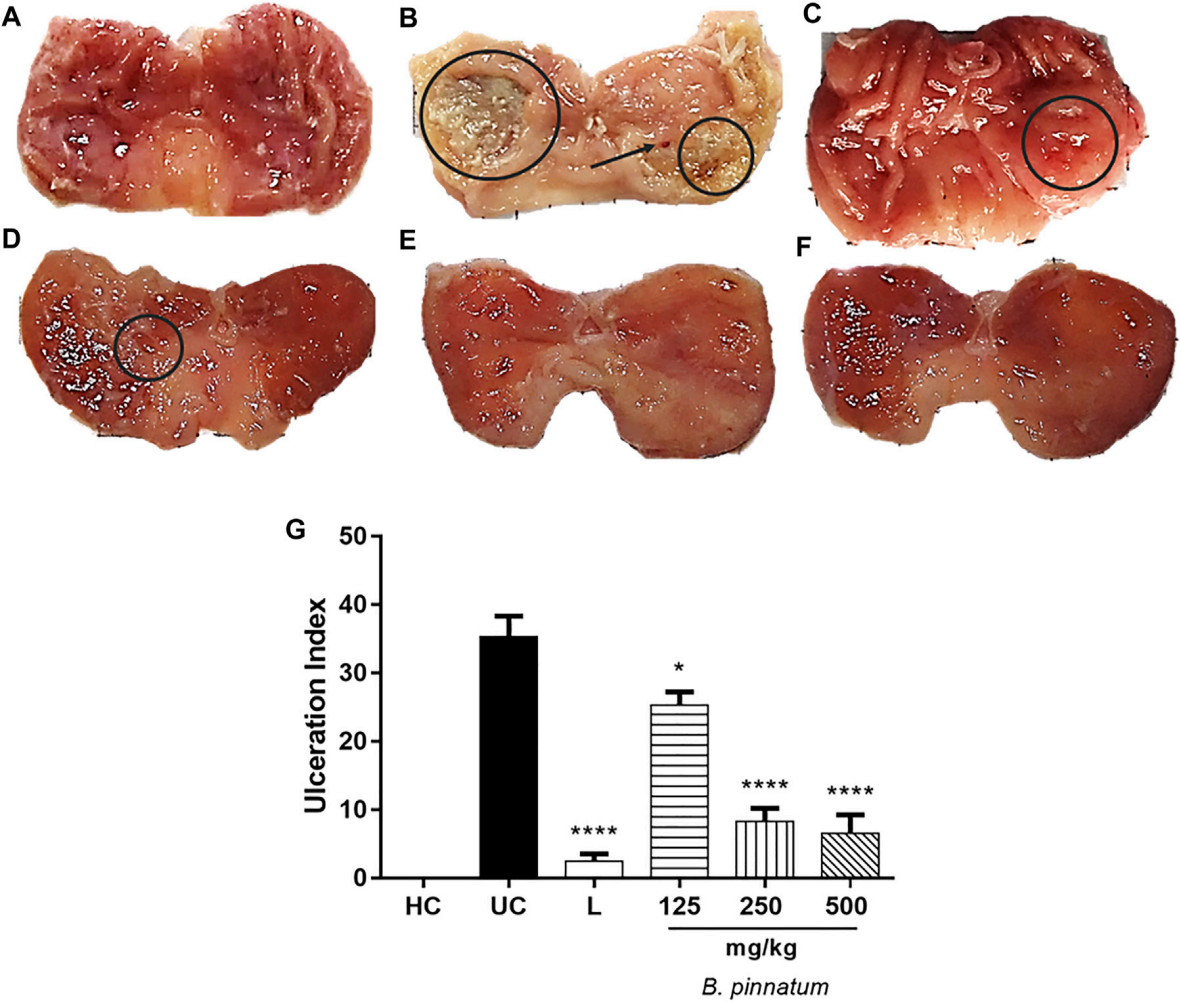
Effect of treatment with *B. pinnatum* leaf extract (125, 250, and 500 mg/kg) on the acetic acid–induced chronic gastric ulcer model in rats **(A)** healthy; **(B)** gastric lesion control; **(C)** lansoprazole 30 mg/kg; **(D)** *B. pinnatum* 125 mg/kg; **(E)** *B. pinnatum* 250 mg/kg; **(F)** *B. pinnatum* 500 mg/kg. **(G)** Results expressed as mean ± standard mean error (*n* = 6). ANOVA and Dunnett post-test were used to calculate the statistical significance, *p < 0.05, **p < 0.01, and ****p < 0.0001 vs. ulcerated control. Healthy control, HC; ulcerated control, UC; lansoprazole (30 mg/kg), L.

Our results showed that the 80% acetic acid instillation in the subserosal layer caused a chronic and severe ulcer with extensive eroded areas in the deep layers of the gastric mucosa (Figure 3B). Treatment with *B. pinnatum* extract at doses of 125 mg/kg (*p* < 0.05), 250 mg/kg (*p* < 0.0001), and 500 mg/kg (*p* < 0.0001) and with lansoprazole at 30 mg/kg (*p* < 0.0001) significantly reduced the ulcerated area and ulceration index when compared to the ulcerated control group (Figure 3; Table 2), showing that the treatment with *B. pinnatum* leaf extract could reduce the lesion area (mm) and consequently stimulate the healing process of chronic ulcers provoked by 80% acetic acid.

**TABLE 2:** Effect of treatment with *B. pinnatum* leaf extract (125, 250, and 500 mg/kg) in the lesion area and percent inhibition in the acetic acid–induced chronic gastric ulcer model.

Experimental group	Lesion area (mm)	Inhibition percentage (%)
Ulcerated control	188.20	—
Lansoprazole (30 mg/kg)	17.67****	92.89
*B. pinnatum* (125 mg/kg)	111.80*	28.13
*B. pinnatum* (250 mg/kg)	54.80****	76.46
*B. pinnatum* (500 mg/kg)	47.40****	81.33

Results expressed as mean ± standard deviation (*n* = 6). ANOVA and Dunnett post-test were used to calculate the statistical significance, *p < 0.05, **p < 0.01 and ****p < 0.0001 vs. ulcerated control.

#### Effect of *Bryophyllum pinnatum* Leaf Extract on Nonprotein Sulfhydryls Levels

**FIGURE 4:**
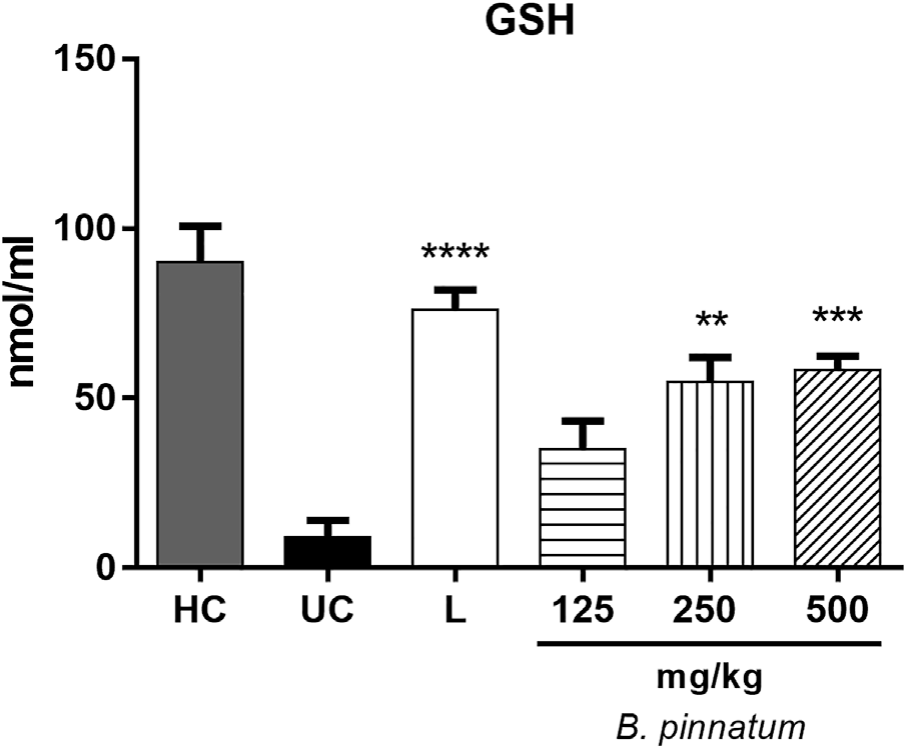
Effect of treatment with *B. pinnatum* leaf extract (125, 250, and 500 mg/kg) on GSH levels in the acetic acid–induced chronic gastric ulcer model in rats. Results expressed as mean ± standard mean error (*n* = 6). ANOVA and Tukey post-test were used to calculate the statistical significance, **p < 0.01, ***p < 0.001, ****p < 0.0001 vs. ulcerated control. Healthy control, HC; ulcerated control, UC; lansoprazole (30 mg/kg), L.

In Figure 4, it is observed that the GSH levels in the healthy gastric tissue are elevated when compared with the ulcerated control group; this occurs because the 80% acetic acid instillation in the subserosal layer resulted in a decrease in the basal GSH levels. Treatment with *B. pinnatum* leaf extract at doses of 250 mg/kg (*p* < 0.01) and 500 mg/kg (*p* < 0.001) could increase the GSH levels in the gastric tissue when compared with the ulcerated control group (Figure 4). In addition, the treatment with the drug lansoprazole 30 mg/kg (*p* < 0.0001) also significantly increase the GSH levels in the gastric tissue when compared to the ulcerated control group (Figure 4).

**FIGURE 5:**
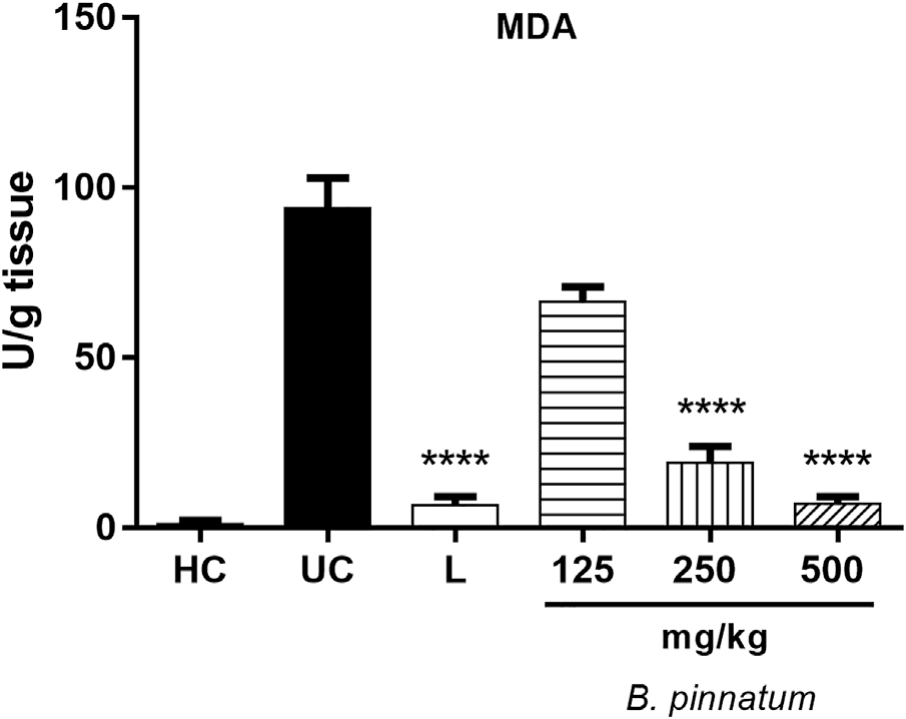
Effect of treatment with *B. pinnatum* leaf extract (125, 250, and 500 mg/kg) on MDA levels in the acetic acid–induced chronic gastric ulcer model in rats. Results expressed as mean ± standard mean error (*n* = 6). ANOVA and Tukey post-test were used to calculate the statistical significance, ****p < 0.0001 vs. ulcerated control. Healthy control, HC; ulcerated control, UC; lansoprazole (30 mg/kg), L.

The 80% acetic acid instillation in the subserosal layer resulted in an increase in MDA levels in the gastric tissue of the ulcerated control group (Figure 5). Treatment with *B. pinnatum* leaf extract at doses of 250 mg/kg (*p* < 0.0001) and 500 mg/kg (*p* < 0.0001) could significantly reduce the MDA levels when compared to the ulcerated control group (Figure 5). Treatment with the drug lansoprazole 30 mg/kg (*p* < 0.0001) also significantly reduced the MDA levels in the gastric tissue when compared to the ulcerated control group (Figure 5).

#### Effect of *Bryophyllum pinnatum* Leaf Extract on Myeloperoxidase Activity

**FIGURE 6:**
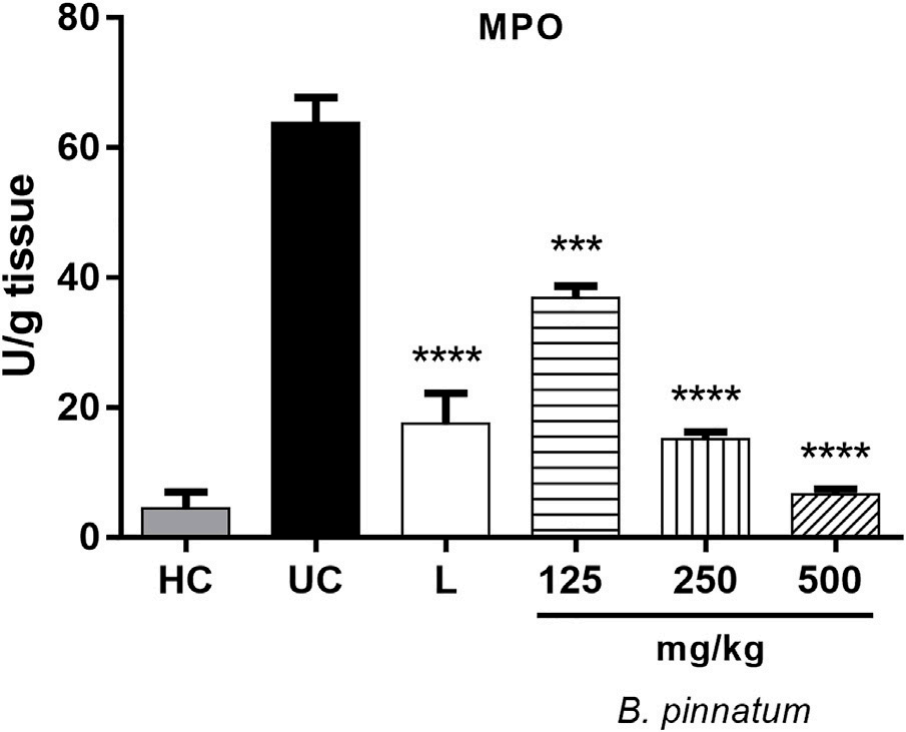
Effect of treatment with *B. pinnatum* leaf extract (125, 250, and 500 mg/kg) on MPO enzyme activity in the acetic acid–induced chronic gastric ulcer model in rats. Results expressed as mean ± standard mean error (*n* = 6). ANOVA and Tukey post-test were used to calculate the statistical significance, ***p < 0.001, ****p < 0.0001 vs. ulcerated control. Healthy control, HC; ulcerated control, UC; lansoprazole (30 mg/kg), L.

Figure 6 shows that the 80% acetic acid instillation in the subserosal layer provoked an increase in the MPO enzyme activity. Treatment with *B. pinnatum* leaf extract at doses of 125 mg/kg (*p* < 0.001), 250 mg/kg (*p* < 0.0001), and 500 mg/kg (*p* < 0.0001) significantly reduced the MPO enzyme activity compared to the ulcerated control group **(** Figure 6). The treatment with lansoprazole at 30 mg/kg (*p* < 0.0001) significantly reduced the MPO enzyme activity in this assay.

#### Effect of *Bryophyllum pinnatum* Leaf Extract on Interleukin-1β, Tumor Necrosis Factor-α, and Interleukin 10 Levels

**FIGURE 7:**
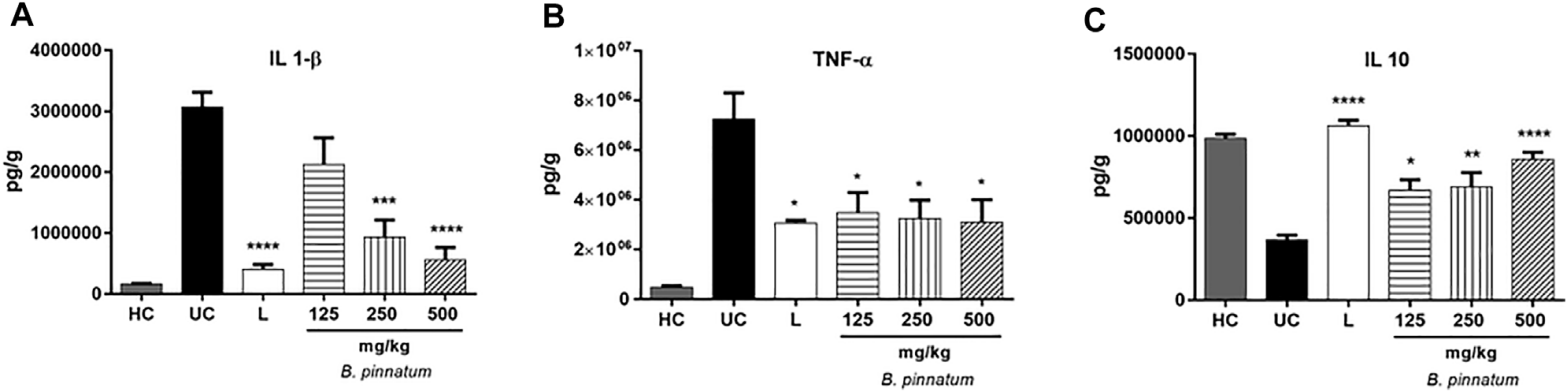
Effect of treatment with *B. pinnatum* leaf extract (125, 250, and 500 mg/kg) on IL-1β, TNF-α, and IL-10 levels in the acetic acid–induced chronic gastric ulcer model in rats. Results expressed as mean ± standard mean error (*n* = 6). ANOVA and Tukey post-test were used to calculate the statistical significance, *p < 0.05, **p < 0.01, ***p < 0.001, ****p < 0.0001 vs. ulcerated control. Healthy control, HC; ulcerated control, UC; lansoprazole (30 mg/kg), L.

The 80% acetic acid instillation in the subserosal layer elevated IL-1β (Figure 7A) and TNF-α (Figure 7B) levels in the gastric tissue. Treatment with *B. pinnatum* leaf extract at doses of 250 mg/kg (*p* < 0.001) and 500 mg/kg (*p* < 0.0001) could significantly reduce IL-1β when compared to the ulcerated control group (Figure 7A). It was also observed that treatment with *B. pinnatum* leaf extract at doses of 125 mg/kg (*p* < 0.05), 250 mg/kg (*p* < 0.05), and 500 mg/kg (*p* < 0.05) could reduce the TNF-α levels in the gastric tissue (Figure 7B). Lansoprazole treatment at a dose of 30 mg/kg could also significantly reduce the IL-1β (*p* < 0.0001) and TNF-α (*p* < 0.05) levels in the gastric mucosa (Figures 7A,B, respectively). Figure 7C shows that the 80% acetic acid instillation in the subserosal layer reduced the IL-10 levels in the ulcerated control. The treatment with *B. pinnatum* at doses of 125 mg/kg (*p* < 0.05), 250 mg/kg (*p* < 0.01), and 500 mg/kg (*p* < 0.0001) significantly increased the IL-10 levels compared to the ulcerated control group (Figure 7C). Lansoprazole treatment at a dose of 30 mg/kg (*p* < 0.0001) could significantly increase the IL-10 levels in the gastric tissue (Figure 7C).

#### Histopathology

**FIGURE 8:**
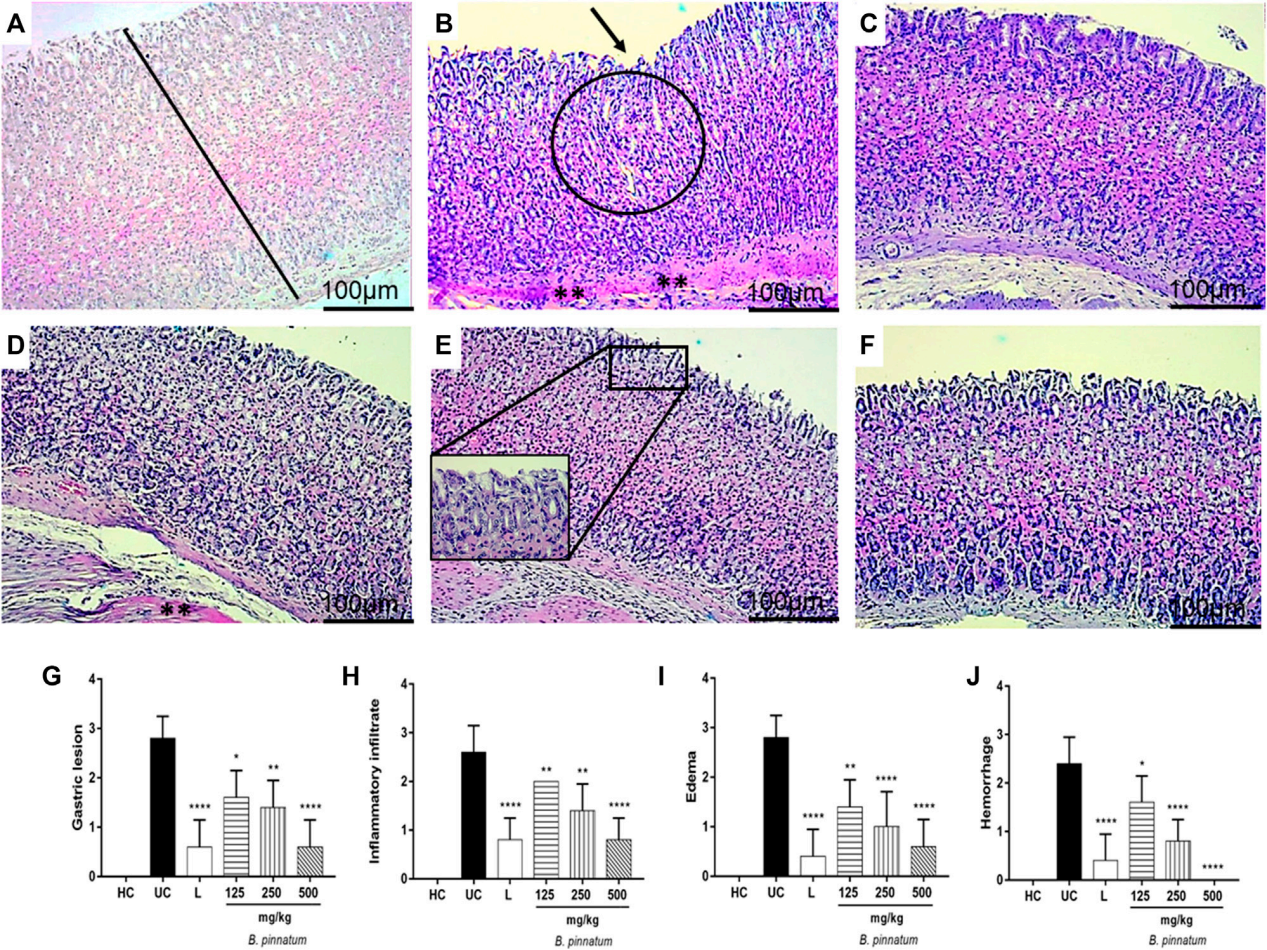
Effect of treatment with *B. pinnatum* leaf extract (125, 250, and 500 mg/kg) in the histology of acetic acid–induced chronic gastric ulcer model in rats. Histopathological characteristics of the gastric tissue of rats, showing the cut of the stomach in the transverse direction. The H&E stained slides were visualized under a bright field microscope with ×10 magnification. **(A)** Healthy control; **(B)** ulcerated control; **(C)** lansoprazole; **(D)** *B. pinnatum* 125 mg/kg; **(E)** *B. pinnatum* 250 mg/kg; **(F)** *B. pinnatum* 500 mg/kg. The bar indicates gastric mucosa without changes; circle: necrotic lesions; one white arrow: hemorrhage points; one black arrow: distraction of the surface epithelium; two asterisks: intense inflammatory infiltrate; **(G)** gastric lesion; **(H)** inflammatory infiltrate; **(I)** edema; **(J)** hemorrhage. Data expressed as mean ± standard mean error (*n* = 5). Mann–Whitney used to calculate statistical significance, *p < 0.05, **p < 0.01, ****p < 0.0001 vs. ulcerated control. Healthy control, HC; ulcerated control, UC; lansoprazole, L.

Figure 8A that pathological changes were not observed in the healthy control group stomachs, as indexed by a semiquantitative score system. However, the 80% acetic acid instillation in the subserosal layer provoked the presence of erosions in the superficial layer and in the deeper layers of the gastric mucosa (Figure 8B). The presence of interruption of the superficial epithelium and also the deeper layers, in addition to inflammatory infiltrate, edema, and hemorrhage, were observed in the ulcerated control group (Figure 8B). Treatment with *B. pinnatum* leaf extract at a dose of 125 mg/kg showed necrotic lesion, hemorrhage points, and intense inflammatory infiltrate (Figure 8D). Treatment with *B. pinnatum* leaf extract at doses of 250 mg/kg (Figure 8E) and 500 mg/kg (Figure 8F) and with lansoprazole at 30 mg/kg (Figure 8C) showed an ulcer healing process with a slight interruption of the superficial epithelium and a slight presence of edema and leukocyte infiltrate. Hemorrhaging was not observed in the treatment with *B. pinnatum* leaf extract at a dose of 500 mg/kg and with lansoprazole 30 mg/kg. Treatment with *B. pinnatum* leaf extract at doses of 250 mg/kg (Figure 8E) and 500 mg/kg (Figure 8F) and with lansoprazole at 30 mg/kg (Figure 8C) could preserve the mucosal and submucosal structural architecture (crypts and gastric glands) when compared to the ulcerated control group.

#### Immunohistochemical Staining of Superoxide Dismutase, Cyclooxygenase-2, and Nuclear Factor-Kappa B-p65

**FIGURE 9:**
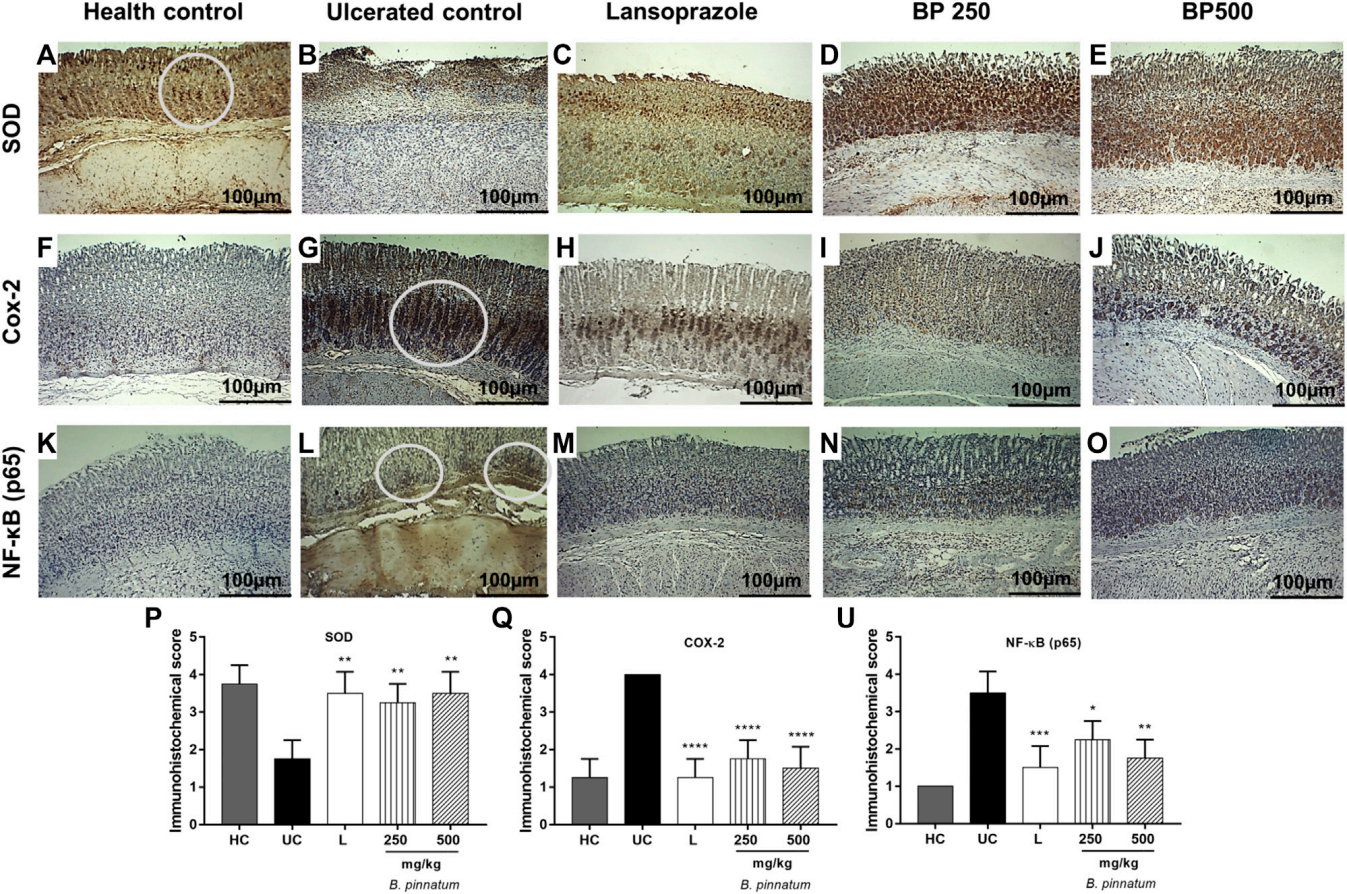
Immunohistochemical analysis of SOD, COX-2, and NF-κB (p65) in rat gastric tissue, showing the cut of the stomach in the transverse direction. **(A–K)** Healthy control; **(B–L)** ulcerated control; **(C–M)** lansoprazole; **(D–N)** *B. pinnatum* 250 mg/kg; **(E–O)** *B. pinnatum* 500 mg/kg; **(P)** SOD immunohistochemical score; **(Q)** COX-2 immunohistochemical score; **(U)** NF-κB (p65) immunohistochemical score. The circle indicates strong brown region. Data expressed as mean ± standard mean error (*n* = 5). Mann–Whitney used to calculate statistical significance, *p < 0.05, **p < 0.01, ***p < 0.001, ****p < 0.0001 vs. ulcerated control. Healthy control, HC; ulcerated control, UC; lansoprazole, L.

Immunohistochemistry analysis for SOD revealed a strong brown (circle) in gastric tissue cells observed in the healthy control group (Figure 9A) and a weak brown in the ulcerated control group (Figure 9B). Treatment with *B. pinnatum* leaf extract at doses of 250 mg/kg (*p* < 0.01) and 500 mg/kg (*p* < 0.01) and lansoprazole upregulated (*p* < 0.01) the expression of this marker in the gastric tissue when compared with the ulcerated gastric control (Figures 9C–E, respectively).

Figures 9G,L show that 80% acetic acid instillation in the subserosal layer resulted in upregulated expression of the COX-2 and NF-κB (p65) in the gastric tissue of the ulcerated control group, observed as strong brown (circle), while treatment with *B. pinnatum* leaf extract at doses of 250 mg/kg (*p* < 0.0001 and *p* < 0.05, respectively, for these markers—Figures 9I,N) and 500 mg/kg (*p* < 0.0001 and *p* < 0.01, respectively, for these markers—Figures 9J,O) downregulated the expression, observed as weak brown. The treatment with lansoprazole (*p* < 0.0001 and *p* < 0.001, respectively—Figures 9H,M) also downregulated the expression of these markers, observed as weak brown.

### Investigating the Gastroprotective Activity of Quercetin 3-*O*-α-L-Arabinopyranosyl-(1→2)-*O*-α-L-Rhamnopyranoside (Bp1) in Gastric Lesions Models

#### Macroscopic Stomach Lesion Assessment

**FIGURE 10:**
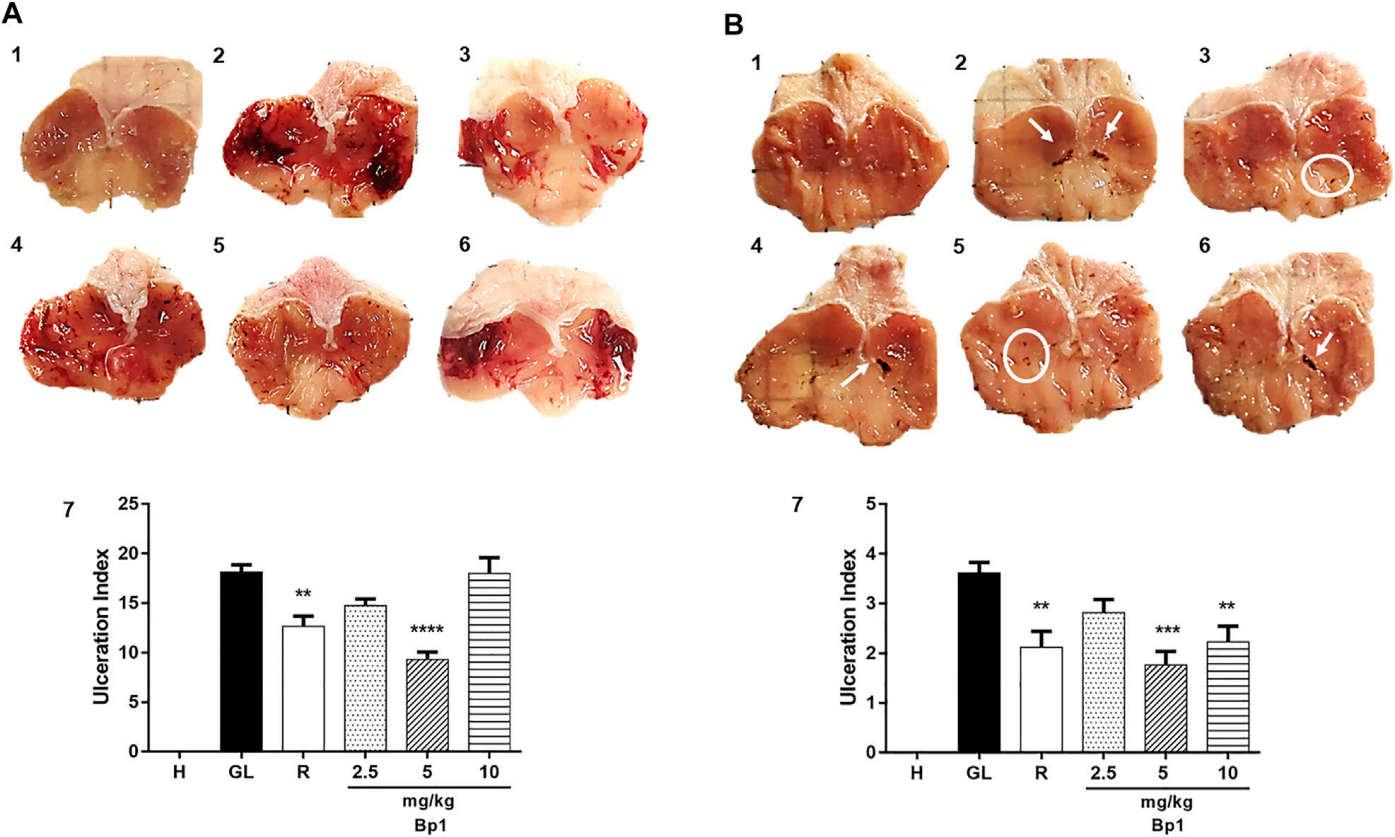
Effect of pretreatment with Bp1 (2.5, 5, and 10 mg/kg) on the macroscopic appearance of the gastric mucosa in ethanol-induced **(A)** and indomethacin-induced **(B)** gastric mucosal lesions in mice. **(A)** Ethanol-induced: 1) healthy; 2) gastric lesion control; 3) ranitidine, 50 mg/kg; 4) Bp1, 2.5 mg/kg; 5) Bp1, 5 mg/kg; 6) Bp1, 10 mg/kg; 7) ulceration index. **(B)** Indomethacin induced: 1) healthy; 2) gastric lesion control; 3) ranitidine, 50 mg/kg; 4) Bp1, 2.5 mg/kg; 5) Bp1, 5 mg/kg; 6) Bp1, 10 mg/kg; 7) ulceration index. Results are expressed as mean ± standard mean error (*n* = 7). ANOVA and Dunnett post-test were used to calculate the statistical significance, **p < 0.01, ***p < 0.001, and ****p < 0.0001 vs. gastric lesion control. Healthy, H; gastric lesion control, GL; ranitidine (50 mg/kg), R.

Figure 10A2 shows that the oral administration of absolute ethanol (0.5 ml/kg) provoked intense lesions in the gastric mucosa with hemorrhagic erosions in the gastric lesion control. The pretreatment with quercetin 3-*O*-α-L-arabinopyranosyl-(1→2)-*O*-α-L-rhamnopyranoside (Bp1) at a dose of 5 mg/kg (*p* < 0.0001) and with ranitidine at 50 mg/kg (*p* < 0.01) significantly reduced the lesions and the ulceration index when compared to the gastric lesion control (Figure 10A 0.5); Table 3 shows that the inhibition percentages and that the pretreatment with quercetin 3-*O*-α-L-arabinopyranosyl-(1→2)-*O*-α-L-rhamnopyranoside (Bp1) and ranitidine could protect the gastric mucosa from developing lesions induced by ethanol.

**TABLE 3:** Effect of pretreatment with Bp1 (2.5, 5, and 10 mg/kg) in the lesion area and percent inhibition in the ethanol-induced model.

Experimental group	Lesion area (mm)	Inhibition percentage (%)
Ulcerated control	37.57 ± 3.46	—
Ranitidine (50 mg/kg)	25.83 ± 5.64**	30.24
Bp1 (2.5 mg/kg)	29.86 ± 3.85	18.65
Bp1 (5 mg/kg)	18.86 ± 4.30****	48.67
Bp1 (10 mg/kg)	36.29 ± 8.46	0.66

Results expressed as mean ± standard deviation (*n* = 7). ANOVA and Dunnett post-test were used to calculate the statistical significance, **p < 0.01 and ****p < 0.0001 vs. gastric lesion control.

Oral administration of indomethacin (40 mg/kg) caused gastric lesion in the gastric lesion control (Figure 10B2 **)**. Pretreatment with quercetin 3-*O*-α-L-arabinopyranosyl-(1→2)-*O*-α-L-rhamnopyranoside (Bp1) at doses of 5 mg/kg (*p* < 0.001) and 10 mg/kg (*p* < 0.01) and with ranitidine at 50 mg/kg (*p* < 0.01) could significantly reduce the ulceration index when compared to the gastric lesion control. It is possible to observe the inhibition percentages in Table 4, and that the pretreatment with quercetin 3-*O*-α-L-arabinopyranosyl-(1→2)-*O*-α-L-rhamnopyranoside (Bp1) and ranitidine could prevent the gastric mucosa from developing lesions; the results for this are expressed as mean ± standard deviation, (*n* = 7). ANOVA and Dunnett post-test were used to calculate the statistical significance, **p < 0.01 and ***p < 0.001 vs. gastric lesion control.

**TABLE 4:** Effect of pretreatment with Bp1 (2.5, 5, and 10 mg/kg) in the lesion area and percent inhibition in the indomethacin-induced model.

Experimental group	Lesion area (mm)	Inhibition percentage (%)
Ulcerated control	7.29 ± 1.11	—
Ranitidine (50 mg/kg)	4.33 ± 1.63**	41.43
Bp1 (2.5 mg/kg)	5.71 ± 1.38	22.09
Bp1 (5 mg/kg)	3.57 ± 1.51***	51.38
Bp1 (10 mg/kg)	4.57 ± 1.72**	38.39

Results expressed as mean ± standard deviation (*n* = 7). ANOVA and Dunnett post-test were used to calculate the statistical significance, **p < 0.01 and ***p < 0.001 vs. gastric lesion control.

#### Effect of Quercetin 3-*O*-α-L-Arabinopyranosyl-(1→2)-*O*-α-L-Rhamnopyranoside (Bp1) Nonprotein Sulfhydryls Levels

**FIGURE 11:**
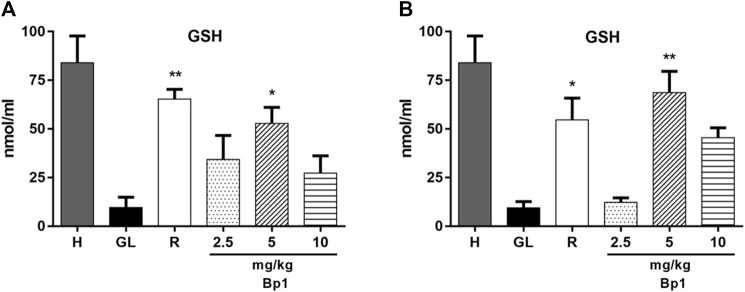
Effect of pretreatment with Bp1 (2.5, 5, and 10 mg/kg) on GSH levels in the gastric mucosa homogenate of lesions in mice **(A)** ethanol-induced and **(B)** indomethacin-induced. Results expressed as mean ± standard mean error (*n* = 7). ANOVA and Tukey post-test were used to calculate the statistical significance, *p < 0.05 and **p < 0.01 vs. gastric lesion control. Healthy, H; gastric lesion control, GL; ranitidine (50 mg/kg), R.

Figures 11A,B show that the oral administration of ethanol (0.5 ml/kg) and indomethacin (40 mg/kg) in mice provoked a decrease in GSH levels in the gastric tissue. The pretreatment with quercetin 3-*O*-α-L-arabinopyranosyl-(1→2)-*O*-α-L-rhamnopyranoside (Bp1) at a dose of 5 mg/kg significantly elevated the GSH levels in the gastric tissue in the ethanol- (*p* < 0.5) (Figure 11A) and indomethacin-induced (*p* < 0.01) (Figure 11B) assays when compared with the gastric lesion control. The pretreatment with ranitidine (50 mg/kg) also significantly elevated the GSH levels in the gastric tissue in the ethanol- (*p* < 0.01) (Figure 11A) and indomethacin-induced (*p* < 0.5) (Figure 11B) assays when compared with the gastric lesion control.

**FIGURE 12:**
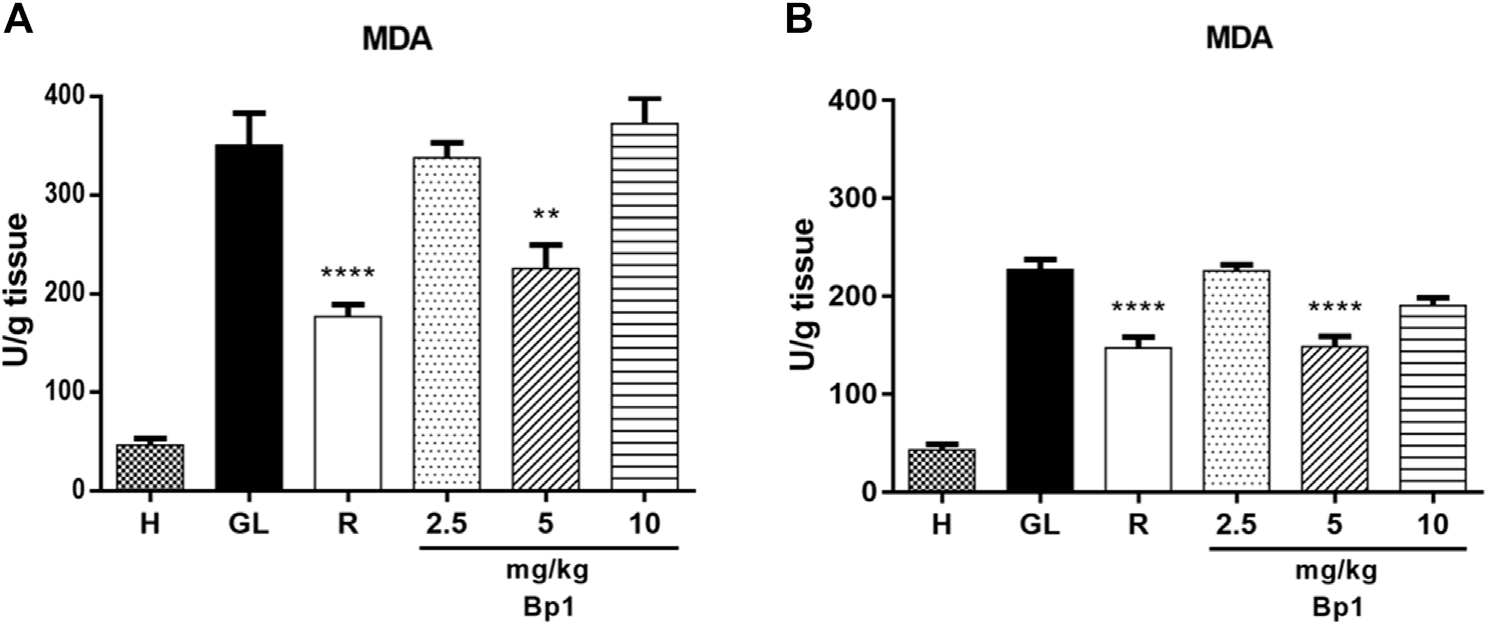
Effect of pretreatment with Bp1 (2.5, 5, and 10 mg/kg) on MDA levels in the gastric mucosa homogenate of lesions in mice **(A)** ethanol-induced and **(B)** indomethacin-induced. Results expressed as mean ± standard mean error (*n* = 7). ANOVA and Tukey post-test were used to calculate the statistical significance, **p < 0.01 and ****p < 0.0001 vs. gastric lesion control. Healthy, H; gastric lesion control, GL; ranitidine (50 mg/kg), R.

The oral administration of ethanol (0.5 ml/kg) and indomethacin (40 mg/kg) in mice provoked an increase in MDA levels in the gastric tissue (Figures 12A,B). The pretreatment with quercetin 3-*O*-α-L-arabinopyranosyl-(1→2)-*O*-α-L-rhamnopyranoside (Bp1) at a dose of 5 mg/kg (*p* < 0.01) significantly reduced the MDA levels in the gastric tissue when compared to the gastric lesion control in the ethanol-induced assay (Figure 12A). The pretreatment with quercetin 3-*O*-α-L-arabinopyranosyl-(1→2)-*O*-α-L-rhamnopyranoside (Bp1) at a dose of 5 mg/kg (*p* < 0.0001) also significantly reduced the MDA levels in the gastric mucosa in the indomethacin-induced assay (Figure 12B). The pretreatment with ranitidine 50 mg/kg significantly reduced the MDA levels in the gastric tissue in the ethanol- (*p* < 0.0001) and indomethacin-induced (*p* < 0.0001) assays (Figures 12A,B, respectively).

#### Effect of Quercetin 3-*O*-α-L-Arabinopyranosyl-(1→2)-*O*-α-L-Rhamnopyranoside (Bp1) on Myeloperoxidase Activity

**FIGURE 13:**
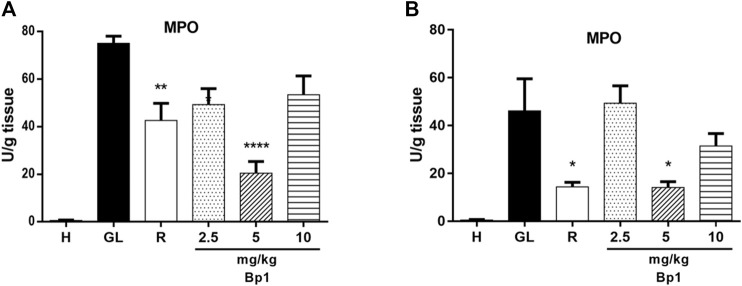
Effect of pretreatment with Bp1 (2.5, 5, and 10 mg/kg) on MPO enzyme activity in the gastric mucosa homogenate of lesions in mice **(A)** ethanol-induced and **(B)** indomethacin-induced. Results expressed as mean ± standard mean error (*n* = 7). ANOVA and Tukey post-test were used to calculate the statistical significance, *p < 0.05, **p < 0.01, and ****p < 0.0001 vs. gastric lesion control. Healthy, H; gastric lesion control, GL; ranitidine (50 mg/kg), R.

Figures 13A,B show that the oral administration of ethanol (0.5 ml/kg) and indomethacin (40 mg/kg) in mice caused an increase in the MPO enzyme activity in the gastric tissue. The pretreatment with quercetin 3-*O*-α-L-arabinopyranosyl-(1→2)-*O*-α-L-rhamnopyranoside **(**Bp1) at a dose of 5 mg/kg (*p* < 0.0001) significantly reduced the MPO enzyme activity in the gastric tissue when compared to the gastric lesion control in the ethanol-induced assay (Figure 13A). In addition, pretreatment with quercetin 3-*O*-α-L-arabinopyranosyl-(1→2)-*O*-α-L-rhamnopyranoside (Bp1) at a dose of 5 mg/kg (*p* < 0.05) also significantly reduced the MPO enzyme activity in the gastric mucosa when compared to the gastric lesion control in the indomethacin-induced assay (Figure 13B). The pretreatment with ranitidine 50 mg/kg significantly reduced the MPO enzyme activity in the gastric mucosa in the ethanol- (*p* < 0.01) and indomethacin-induced (*p* < 0.05) assays (Figures 13A,B, respectively).

#### Effect of Quercetin 3-*O*-α-L-Arabinopyranosyl-(1→2)-*O*-α-L-Rhamnopyranoside (Bp1) Pretreatment on Interleukin-1β, Tumor Necrosis Factor-α, and IL-10 Levels

**FIGURE 14:**
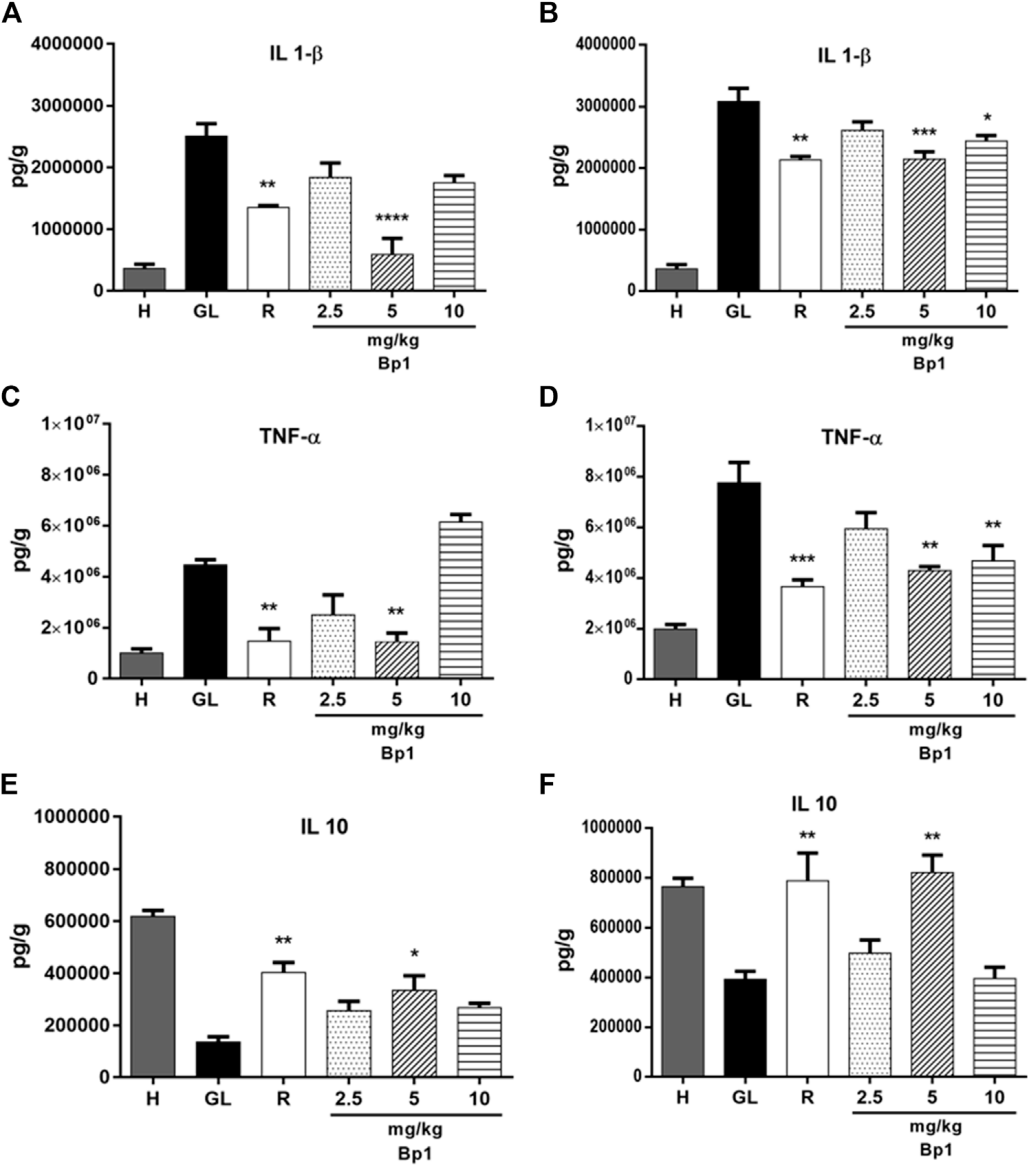
Effect of pretreatment with Bp1 (2.5, 5, and 10 mg/kg) on IL-1β, TNF-α, and IL-10 levels in the gastric mucosa homogenate of lesions in mice **(A–E)** ethanol-induced and **(B–F)** indomethacin-induced. Results expressed as mean ± standard mean error (*n* = 7). ANOVA and Tukey post-test were used to calculate the statistical significance, *p < 0.05, **p < 0.01, ***p < 0.001, and ****p < 0.0001 vs. gastric lesion control. Healthy, H; gastric lesion control, GL; ranitidine (50 mg/kg), R.

The oral administration of ethanol (0.5 ml/kg) and indomethacin (40 mg/kg) in mice elevated IL-1β and TNF-α levels in the gastric tissue (Figures 14A–D). The pretreatment with Bp1 at a dose of 5 mg/kg significantly reduced the IL-1β (*p* < 0.0001) and TNF-α (*p* < 0.01) levels in the gastric mucosa when compared to the gastric lesion control in the ethanol-induced assay (Figures 14A,C, respectively). In addition, the pretreatment with Bp1 at a dose of 5 mg/kg significantly reduced the IL-1β (*p* < 0.001) and TNF-α (*p* < 0.01) levels and at a dose of 10 mg/kg also significantly reduced the IL-1β (*p* < 0.05) and TNF-α (*p* < 0.01) levels in the gastric tissue when compared to the gastric lesion control in the indomethacin-induced assay (Figures 14B,D, respectively). The pretreatment with ranitidine at 50 mg/kg significantly reduced the IL-1β (*p* < 0.01 in both models) and TNF-α (*p* < 0.1 in the ethanol-induced assay and *p* < 0.001 in the indomethacin-induced assay) levels in the gastric tissue when compared to the gastric lesion control.

The oral administration of ethanol (0.5 ml/kg) and indomethacin (40 mg/kg) also reduced the IL-10 levels in the gastric tissue (Figures 14E,F). Pretreatment with Bp1 at a dose of 5 mg/kg significantly elevated the IL-10 levels in the gastric tissue when compared to the gastric lesion control in the ethanol- (*p* < 0.05) and indomethacin-induced (*p* < 0.01) assays (Figures 14E,F, respectively). The pretreatment with ranitidine at a dose of 50 mg/kg also significantly elevated the IL-10 levels in the gastric tissue when compared to the gastric lesion control in the ethanol- (*p* < 0.01) and indomethacin-induced (*p* < 0.01) assays (Figures 14E,F, respectively).

#### Histopathology

**FIGURE 15:**
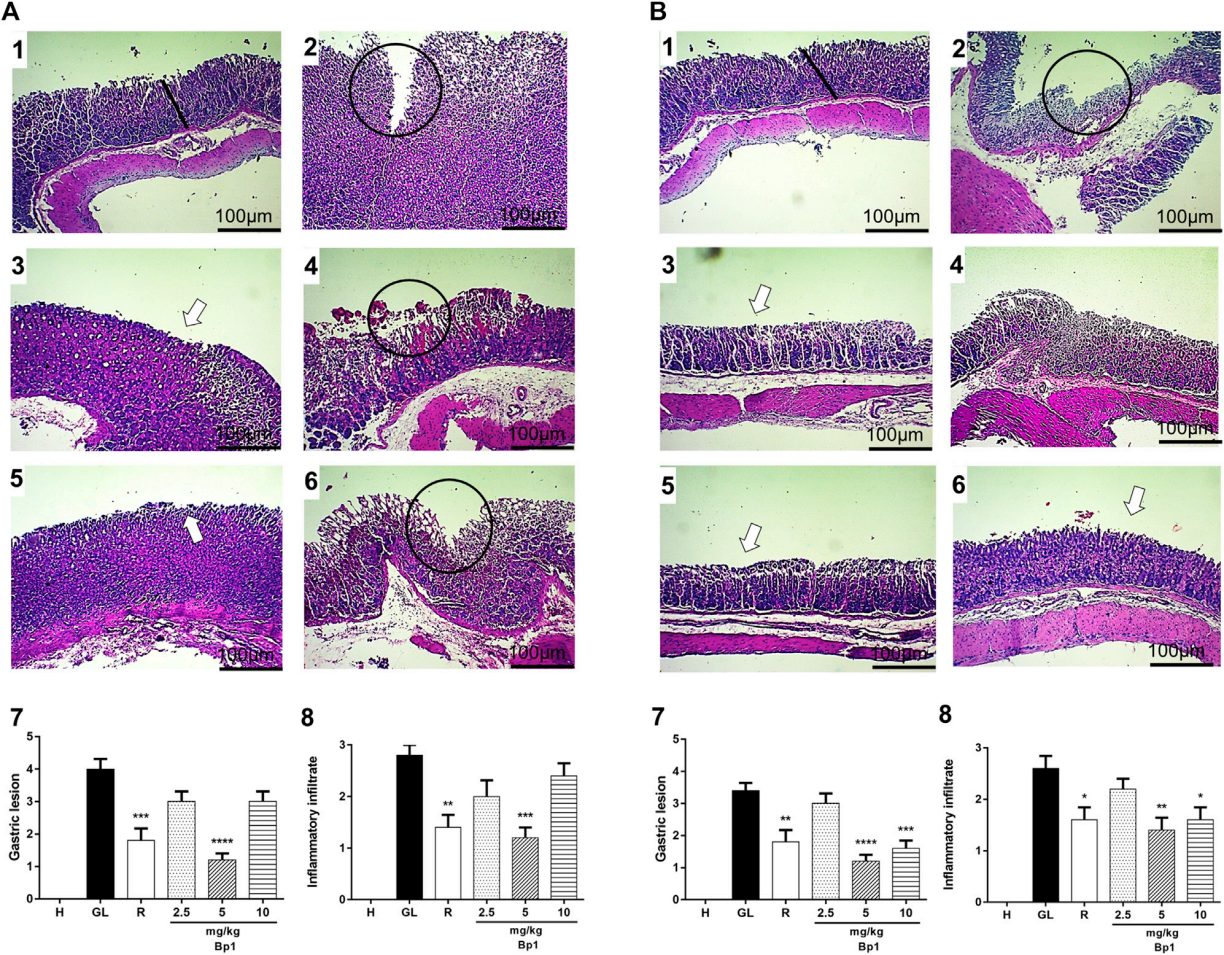
Effect of pre-treatment with Bp1 (2.5, 5, and 10 mg/kg) on the histology of ethanol-induced **(A)** and indomethacin-induced **(B)** gastric mucosal lesions in mice. Histopathological characteristics of the gastric tissue of rats, showing the cut of the stomach in the transverse direction. **(A)** Ethanol-induced: (1) Healthy; (2) Gastric lesion control; (3) Ranitidine, 50 mg/kg; (4) **Bp1** 2.5 mg/kg; (5) **Bp1** 5 mg/kg; (6) **Bp1** 10 mg/kg; (7) Gastric lesion score; (8) Inflammatory infiltrate score. **(B)** Indomethacin-induced: (1) Healthy; (2) Gastric lesion control; (3) Ranitidine, 50 mg/kg; (4) **Bp1** 2.5 mg/kg; (5) **Bp1** 5 mg/kg; (6) **Bp1** 10 mg/kg; (7) Gastric lesion score; (8) Inflammatory infiltrate. The bar indicates gastric mucosa without changes; Circle: severe lesion with distraction of the surface epithelium; White arrow: recovered mucosa. Results expressed as mean ± standard mean error (*n* = 7). Mann–Whitney used to calculate statistical significance, *p < 0.05, **p < 0.01, ***p < 0.001, and ****p < 0.0001 vs. gastric lesion control. Healthy, H; Gastric lesion control, GL; Ranitidine (50 mg/kg), R. gastric mucosal damage in rats.

According to the histopathology assay, the Healthy Group showed intact mucosa without changes (Figures 15A1,B1). However, the oral administration of ethanol and indomethacin provoked a severe lesion in the gastric mucosa with the presence of extensive edema and leukocyte infiltration in the mucosa and submucosa layers (Figures 15A2,B2). The pretreatment with 3-*O*-α-L-arabinopyranosyl-(1→2)-*O*-α-L-rhamnopyranoside (Bp1) at doses of 2.5 and 10 mg/kg showed severe lesions in the mucosa with edema and leukocyte infiltration in the ethanol–gastric lesion model (Figures 15A4,A6), while the pretreatment with BP1 at a dose of 5 mg/kg and with ranitidine at 50 mg/kg reduced the severity of the lesions, presenting a slight interruption of the superficial epithelium with submucosal edema and minimal leukocyte infiltration (Figures 15A3,A5). Pretreatment with Bp1 at a dose of 2.5 mg/kg showed severe injuries in the gastric mucosa with edema and leukocyte infiltration in the indomethacin gastric lesion model (Figures 15B4), while the pretreatment with Bp1 at doses of 5 and 10 mg/kg and with ranitidine at a dose of 50 mg/kg improved lesion severity, presenting a slight interruption of the superficial epithelium with submucosal edema and minimal leukocyte infiltration (Figures 15B3–B6, respectively).

To summarize the results obtained in this work, pretreatment with quercetin 3-*O*-α-L-arabinopyranosyl-(1→2)-*O*-α-L-rhamnopyranoside (Bp1) and the treatment with *B. pinnatum* extract showed gastroprotective and anti-ulcer activity, respectively. In addition to reduced macroscopic damage and reorganization of the mucosal structure by histopathological analysis, these potential pharmacological effects were evidenced by increasing the antioxidant defense system and modulating inflammatory markers (Table 5).

**TABLE 5:** Modulation of antioxidant defense system and inflammatory markers.

Inflammatory markers	*B. pinnatum* (125 mg/kg)	*B. pinnatum* (250 mg/kg)	*B. pinnatum* (500 mg/kg)	Bp1 (2.5 mg/kg)	Bp1 (5 mg/kg)	Bp1 10 (mg/kg)
GSH	N.S	↑	↑	N.S	↑	N.S
MDA	N.S	↓	↓	N.S	↓	N.S
SOD	N.S	↑	↑	—	—	—
MPO	N.S	↓	↓	N.S	↓	N.S
IL-1β	N.S	↓	↓	N.S	↓	↓
TNF-α	↓	↓	↓	N.S	↓	↓
IL-10	↑	↑	↑	N.S	↑	N.S
NF-κB (p65)	N.S	↓	↓	—	—	—
COX-2	N.S	↓	↓	—	—	—

N.S, not statically significant.

## Discussion

In this study, it was observed that treatment with *B. pinnatum* leaf extract could promote gastric healing of chronic ulcers in the gastric tissue of rats induced by 80% acetic acid. In addition to healing the lesion area, treatment with this species could also improve the inflammatory and oxidative stress markers evaluated. It was also observed that the pretreatment with quercetin 3-*O*-α-L-arabinopyranosyl-(1→2)-*O*-α-L-rhamnopyranoside **(**Bp1**)** protected the gastric mucosa of mice against acute lesions caused by ethanol and indomethacin, thus suggesting that this flavonoid had a potential gastroprotective effect. In addition to reducing the lesion area, the pretreatment with this flavonoid significantly improved the oxidative stress and inflammation parameters in the gastric tissue.

The chronic gastric ulcer caused by acetic acid instillation was similar to human ulcers because they develop in the same location and showed an equal degree of severity and chronicity, as well as in the healing process of the affected area (Okabe et al., 2005). The development of ulcers by acetic acid induction occurs due to changes in different factors such as PG production, growth factor, NO, cytokine contents, and mucus production (Tarnawsk, 2005; Kang et al., 2014). The healing of chronic ulcers is a complex process which involves cell migration, proliferation, and replication of epithelial cells near the margins to restore the glandular architecture and granulation tissue angiogenesis at the base of the ulcer (Tarnawsk, 2005; Kang et al., 2014). Corroborating the hypothesis that the *B. pinnatum* leaf extract could stimulate gastric healing, the ulcerated area provoked by acetic acid was significantly reduced by the treatment with this species in a dose-dependent manner. It is possible to note a reduction in the ulcerated area by macroscopic analysis (Figures 3E,F) and by histological analysis (Figures 8E,F) of the lesion of the *B. pinnatum* extract–treated group at doses of 250 and 500 mg/kg. In Figures 3B, 8B, it is possible to observe extensive erosive lesions in the mucous layer and in deeper layers of the gastric wall in the ulcerated control group. In addition, reorganization of the epithelium with healing of the ulcerated area can also be observed macroscopically and histologically in the lansoprazole -treated group at a dose of 30 mg/kg (Figures 3C, 8C).

Quercetin 3-*O*-α-L-arabinopyranosyl-(1→2)-*O*-α-L-rhamnopyranoside (Bp1) is considered the major compound and the marker for *B. pinnatum* (Nascimento et al., 2018; Fernandes et al., 2021) therefore, experiments with Bp1 in gastric lesions induced by ethanol and indomethacin were conducted in order to assess whether the effect of the extract was related to the presence of this compound. The induction of acute gastric lesions by ethanol and indomethacin could cause intense damage to the gastric tissue in the form of ulcerative lesions in the mucosa layer (Tu et al., 2017; Czekaj et al., 2018; Kangwan et al., 2019; Ugwah et al., 2019).

Ethanol induces acute gastric lesions by increasing lipid peroxidation and oxidative stress (Czekaj et al., 2018), which consequently leads to developing injuries in the mucosa layer with increasing MDA, inflammatory cytokine, and NO production levels, as well as an increase in the MPO enzyme activity. Ethanol decreases the PGE2 and antioxidant enzyme activity levels, reduces the secretion of bicarbonate and mucus generation, in addition to producing excessive (reactive oxygen species) ROS, disturbs the gastric microcirculation, and provokes lesions on the epithelial cells, causing a rupture in mucous cell membranes and cytotoxic effects (Tu et al., 2017).

Oral administration of indomethacin can cause serious macroscopic damage to the gastric mucosa layer, such as a loss of normal color, mild hemorrhaging, moderate edema, and severe mucosal disruption. Indomethacin weakens the gastric mucosa by inhibiting synthesis of PGs by cyclooxygenase-1 (COX-1). In addition, NSAIDs also act as a prooxidant catalyst and initiate the lipoperoxidation-producing ROS, in turn interfering in the antioxidant system of endogenous cells of the mucosa, inducing leukocyte recruitment, and boosting the inflammatory response (Kangwan et al., 2019; Ugwah et al., 2019).

In this work, it was possible to note that the pretreatment with quercetin 3-*O*-α-L-arabinopyranosyl-(1→2)-*O*-α-L-rhamnopyranoside **(**Bp1**)** at a dose of 5 mg/kg in the ethanol-induced model (Figures 10A, 15A, respectively), while the doses of 5 and 10 mg/kg in the indomethacin-induced model (Figures 10B, 15B, respectively), reduced the ulcerated area by macroscopic analysis and by histological analysis. In Figures 10A, B2, 15A2, B2, it is possible to observe extensive erosive lesions in the mucous layer in the gastric lesion control group in both induced models. In addition, the reorganization of the epithelium can also be macroscopically and histologically observed in the ranitidine-treated group at a dose of 50 mg/kg (Figures 10A, B3, 15A3, B3, respectively).

The acetic acid instillation in the subserosal layer induces a state of stress in the gastric tissue, consequently inducing a chronic gastric ulcer. ROS production and oxidative stress are the major cause for developing and aggravating the ulcer. Furthermore, chronic ulcer induced by acetic acid leads to chronic oxidative stress with decreased SOD activity and GSH levels and increased lipid peroxidation (MDA) levels. It is known that SOD, GSH, and MDA play important roles in protecting the gastric mucosa against oxidative gastric mucosal injury (Kwiecien et al., 2002; Adibhatla et al., 2010).

Endogenous sulfhydryl (SH) plays an important role in maintaining the integrity of the gastric mucosa and elevating the basal concentration of nonprotein sulfhydryl, which is mostly reduced GSH (γ-glutamyl-cysteinyl-glycine), indicating their possible implications for gastroprotection (Nagy et al., 2007). MDA is a reliable parameter of ROS-induced mucosal injury (Kwiecien et al., 2002). SOD is a group of metalloenzymes which catalyze the dismutation of superoxide radical into hydrogen peroxide and oxygen. SOD is the first line of defense inside cells against ROS (Fernández et al., 2009). Therefore, an expression analyses by SOD immunohistochemistry and detecting GSH and MDA levels can reflect and indicate oxidative stress in the tissue. Treatment with *B. pinnatum* extract at the doses of 250 and 500 mg/kg and lansoprazole at a dose of 30 mg/kg stimulated SOD expression (Figures 9C–E, respectively) and the GSH levels (Figure 4) in the gastric tissue, in addition to decreasing the MDA levels (Figure 5).

Oral administration of ethanol and indomethacin also causes a state of oxidative stress in the gastric mucosa, provoking the development of gastric lesions (Karkkainen et al., 2015; Lim et al., 2019). Pretreatment with quercetin 3-*O*-α-L-arabinopyranosyl-(1→2)-*O*-α-L-rhamnopyranoside (Bp1) at a dose of 5 mg/kg and ranitidine at 50 mg/kg could elevate the GSH (Figures 11A,B, respectively) and decrease the MDA (Figures 12A,B, respectively) levels in the gastric mucosa. These results suggest that *B. pinnatum* leaf extract and the isolated quercetin 3-*O*-α-L-arabinopyranosyl-(1→2)-*O*-α-L-rhamnopyranoside (Bp1) are capable of stimulating antioxidant system remodeling in the gastric mucosa during the inflammatory process in this tissue.

MPO activity in the ulcerated gastric tissue induced by acetic acid is higher than normal gastric tissue (Keto et al., 2002). Therefore, MPO is an important indicator of inflammation in the chronic gastric model to observe extensive neutrophil infiltration/aggregation in gastric tissue in this situation (Naito et al., 1998). In this study it was observed that the treatment with *B. pinnatum* leaf extract at all doses and lansoprazole 30 mg/kg were capable of decreasing the MPO activity (Figure 6) and the histological analysis (Figures 8E,F) showed a decrease in the inflammatory infiltrate and edema with the treatment with the doses of 250 and 500 mg/kg and with lansoprazole at 30 mg/kg (Figure 8C).

Oral administration of ethanol and indomethacin can result in high neutrophil infiltration with consequent elevated MPO activity collaborating to develop gastric lesions (Hansson 2006; Raesi et al., 2019). Pretreatment with quercetin 3-*O*-α-L-arabinopyranosyl-(1→2)-*O*-α-L-rhamnopyranoside (Bp1) at a dose of 5 mg/kg and with ranitidine at 50 mg/kg decreased the MPO activity in the gastric mucosa (Figures 13A,B).

Gastric inflammation around the ulcer region stimulates the migration of macrophages and polymorphonuclear cells, and this consequently leads to an increase in the release of pro-inflammatory cytokines and mediators form these cells. Pro-inflammatory cytokines such as TNF-α and IL-1β play an important role in the pathogenesis of gastric ulcer (Raeesi and Eskandari-Ro, 2019). IL-1β and TNF-α levels are elevated and IL-10 levels are reduced in chronic gastric ulcer (Haghazali et al., 2011). The beneficial effect of *B. pinnatum* leaf extract can be seen in the modulation of cytokines, reducing the pro-inflammatory cytokines IL-1β and (Figure 7A) TNF-α levels (Figure 7B) and elevating the IL-10 levels, an important anti-inflammatory cytokine (Figure 7C).

Oral administration of ethanol and indomethacin also elevated the IL-1β and TNF-α levels and reduced the IL-10 levels. Pretreatment with quercetin 3-*O*-α-L-arabinopyranosyl-(1→2)-*O*-α-L-rhamnopyranoside (Bp1) at a dose of 5 mg/kg could reduce the IL-1β and TNF-α levels and elevated the IL-10 levels in the ethanol-induced model (Figures 14A,C,E, respectively). In addition, pretreatment with quercetin 3-*O*-α-L-arabinopyranosyl-(1→2)-*O*-α-L-rhamnopyranoside (Bp1) at doses of 5 and 10 mg/kg reduced the IL-1β and TNF-α levels and elevated the IL-10 levels in the gastric mucosa in the indomethacin-induced model (Figures 14B,D,F, respectively). These results associated with the histological analysis indicate that the *B. pinnatum* leaves and its major flavonoid (Bp1) were capable of modulating the inflammatory process. We hypothesize that quercetin 3-*O*-α-L-arabinopyranosyl-(1→2)-*O*-α-L-rhamnopyranoside (Bp1) showed a cytoprotective effect in the gastric mucosa and that the *B. pinnatum* leaf extract promoted the healing process in this tissue.

TNF-α and other pro-inflammatory cytokines activate NF-κB, and this action stimulates the activation of inducible enzyme isoforms of the inflammatory process such as COX-2. COX-2 is upregulated during the inflammatory process, and this results in an elevated production of pro-inflammatory PGs (Karin et al., 2002; Virginia et al., 2005; Gloire et al., 2006; Kang et al., 2014). Immunohistochemical analysis showed that the treatment with *B. pinnatum* extract at doses of 250 and 500 mg/kg and with lansoprazole (30 mg/kg) could decrease NF-κB (Figures 9M–O, respectively) and COX-2 (Figures 9H–J, respectively) expressions in the gastric tissue in the chronic ulcer model.

ROS are considered as the second messenger to initiate redox-sensitive signal transduction pathway with mitogen-activated protein kinase cascade and is related to NF-κB transcription, acting to control the gene expression of several proinflammatory mediators and provoking inflammatory damage in gastric tissue (Kang and Kim, 2017). ROS also simultaneously stimulates the inhibitor kappa B (IκB) kinase which induces proteasomal breakdown of IκBα and activates NF-κB. NF-κB is a transcription factor which promotes target genes and triggers transcription of inflammatory cytokines and chemokines when linking to κ-β. The NF-κB family of transcription factors show an important role in inflammation (Dambrova et al., 2010). Therefore, inhibiting NF-κB activation may be an effective way to prevent and treat gastric ulcer.

The present study showed that three flavonoids—quercetin 3-*O*-α-L-arabinopyranosyl-(1→2)-*O*-α-L-rhamnopyranoside (Bp1), kaempferol 3-*O*-α-L-arabinopyranosyl-(1→2)-*O*-α-L-rhamnopyranoside (Bp2), and quercetin 3-*O*-α-L-rhamnopyranoside (Bp3)—were quantified in *B. pinnatum* extract, with BP1 being the major compound and the possible active marker. These findings are important for quality control of the raw material obtained from this plant.

Flavonoids are a group of phenolic compounds found in different plant species (Glevitzky et al., 2019). The basic structure of flavonoids consists of 15 carbon atoms, arranged in the form C5-C3-C6 (aryl-propyl-aryl), which corresponds to two aromatic rings linked by a unit of three carbon atoms (Justino, 2017). This class of compounds can act as antioxidants by free radical scavenging mechanism (Dangles et al., 2000), and this can be explained due to a lack of -OH groups on the B-ring (Amic et al., 2003).

It is described in the literature that flavonoids have pharmacological activities, for example, this class can inhibit the proliferation of inflammatory cells and consequently suppress the expressions of inflammatory cytokines. In addition, they can also decrease ROS with a decrease in NO concentration and increase in endogen oxidative enzyme levels (Antonisamy et al., 2016; Wang et al., 2017). Therefore, the results obtained from this study corroborate with previous studies and suggest that the quercetin 3-*O*-α-L-arabinopyranosyl-(1→2)-*O*-α-L-rhamnopyranoside (Bp1) flavonoid isolated from the *B. pinnatum* species may be one of the constituents responsible for the anti-ulcer activity of this species.

Nowadays, the bioactivity of isolated phenolics and phenolic-rich extracts focusing on suppression of chronic diseases has been widely discussed in the literature due to their protective effect in inflammatory diseases with a remarkable capacity because of their multiple inhibitory activities of proinflammatory mediators. Phenolic compounds can be found in many foods and medicinal plants; therefore, researchers have proposed that dietary sources rich in phenolic compounds and/or herbal medicines can be an alternative in treating inflammation and related diseases, with minimal or null adverse side effects or in a more natural, drug-free fashion (Ambriz-Pérez et al., 2016; Shahidi and Yeo, 2018; de Moraes et al., 2020). In this perspective, *B. pinnatum* leaf extract can be a new source of raw material which is rich in phenolics to be applied as food or medicine.

However, in a previous study by Araújo et al. (2018), it was observed that the pretreatment with *B. pinnatum* leaf extract against acute gastric lesions models showed a higher inhibition percentage of gastric lesions when only compared with the pretreatment with the isolated flavonoid used in this study. In addition, the treatment with *B. pinnatum* leaf extract against chronic ulcer gastric model carried out in this study also showed a higher inhibition percentage when compared to pretreatment with the isolated flavonoid. This might suggest that because *B. pinnatum* leaf extract is a complex vegetable matrix and has other substances in its composition, it has better anti-ulcer activity than administering the isolated flavonoid. We hypothesize that the other compounds present in it can act synergistically, potentiating the anti-inflammatory effect and consequently the anti-ulcer property of this species. However, we can suggest that future studies should be carried out with this extract and other isolated compounds present in it, such as pharmacokinetic and pharmacodynamic studies, such that more scientific evidence will be generated to confirm or refute this hypothesis.

## Conclusion

Pretreatment with quercetin 3-*O*-α-L-arabinopyranosyl-(1→2)-*O*-α-L-rhamnopyranoside **(**Bp1**)** and treatment with *B. pinnatum* extract exhibited relevant pharmacological effects in preclinical assays from showing significant gastroprotective and anti-ulcer activities, respectively. These effects were mainly observed through increasing the antioxidant defense system and modulating inflammatory markers. Overall, this work can collaborate to develop a new phytotherapeutic treatment of peptic ulcer disease or as a new source of raw material as a functional food ingredient to be incorporated into food matrices.

## Data Availability

The original contributions presented in the study are included in the article/Supplementary Material; further inquiries can be directed to the corresponding author.
